# Post-fire stabilization of thaw-affected permafrost terrain in northern Alaska

**DOI:** 10.1038/s41598-024-58998-5

**Published:** 2024-04-11

**Authors:** Benjamin M. Jones, Mikhail Z. Kanevskiy, Yuri Shur, Benjamin V. Gaglioti, M. Torre Jorgenson, Melissa K. Ward Jones, Alexandra Veremeeva, Eric A. Miller, Randi Jandt

**Affiliations:** 1 Institute of Northern Engineering, University of Alaska Fairbanks, Fairbanks, AK 99775 USA; 2 College of Engineering and Mines, University of Alaska Fairbanks, Fairbanks, AK 99775 USA; 3 Alaska Ecoscience, Fairbanks, AK 99775 USA; 4 Alfred Wegener Institute Helmholtz Centre for Polar and Marine Research, Potsdam, Germany; 5 Bureau of Land Management Alaska Fire Service, Fort Wainwright, AK 99703 USA; 6 Alaska Fire Science Consortium, University of Alaska Fairbanks, Fairbanks, AK 99775 USA

**Keywords:** Climate sciences, Environmental sciences

## Abstract

In 2007, the Anaktuvuk River fire burned more than 1000 km^2^ of arctic tundra in northern Alaska, ~ 50% of which occurred in an area with ice-rich syngenetic permafrost (Yedoma). By 2014, widespread degradation of ice wedges was apparent in the Yedoma region. In a 50 km^2^ area, thaw subsidence was detected across 15% of the land area in repeat airborne LiDAR data acquired in 2009 and 2014. Updating observations with a 2021 airborne LiDAR dataset show that additional thaw subsidence was detected in < 1% of the study area, indicating stabilization of the thaw-affected permafrost terrain. Ground temperature measurements between 2010 and 2015 indicated that the number of near-surface soil thawing-degree-days at the burn site were 3 × greater than at an unburned control site, but by 2022 the number was reduced to 1.3 × greater. Mean annual ground temperature of the near-surface permafrost increased by 0.33 °C/yr in the burn site up to 7-years post-fire, but then cooled by 0.15 °C/yr in the subsequent eight years, while temperatures at the control site remained relatively stable. Permafrost cores collected from ice-wedge troughs (n = 41) and polygon centers (n = 8) revealed the presence of a thaw unconformity, that in most cases was overlain by a recovered permafrost layer that averaged 14.2 cm and 18.3 cm, respectively. Taken together, our observations highlight that the initial degradation of ice-rich permafrost following the Anaktuvuk River tundra fire has been followed by a period of thaw cessation, permafrost aggradation, and terrain stabilization.

## Introduction

The frequency and severity of northern high latitude tundra fires has increased since the early-2000s relative to historical^1–3^ and paleo-ecological records^4–6^. The increase in burned tundra area is an indicator of climate change impacts in a warming Arctic^7,8^. The increase in tundra fires has been attributed to periods of sustained drought conditions and an increase in lightning activity^9–11^. Increases in tundra fire disturbances exerts strong controls on vegetation^12–17^, carbon and nutrient cycling^18–23^, and permafrost terrain stability^24–27^. Chen et al.^24^ suggested tundra fire disturbance, while representing just 3% of the overall area, was responsible for 11% of the areal extent of all thermokarst detected between 1950 and 2015 across 4700 km^2^ area in arctic Alaska.

Permafrost thaw following northern fires has also become more apparent since the early-2000s^3,16,25–29^. Identifying the processes controlling post-fire thermokarst development in permafrost regions is important because ground thaw threatens to release globally significant amounts of permafrost soil carbon as greenhouse gases^30^. Fire in these regions act as a pulse disturbance mechanism that mobilizes carbon through the combustion of vegetation^31–33^, burning of surface soil organic layers^22^, and, in some cases, the additional release of soil organic carbon through enhanced soil respiration triggered by post-fire permafrost thaw^34,35^.

**Figure 1:**
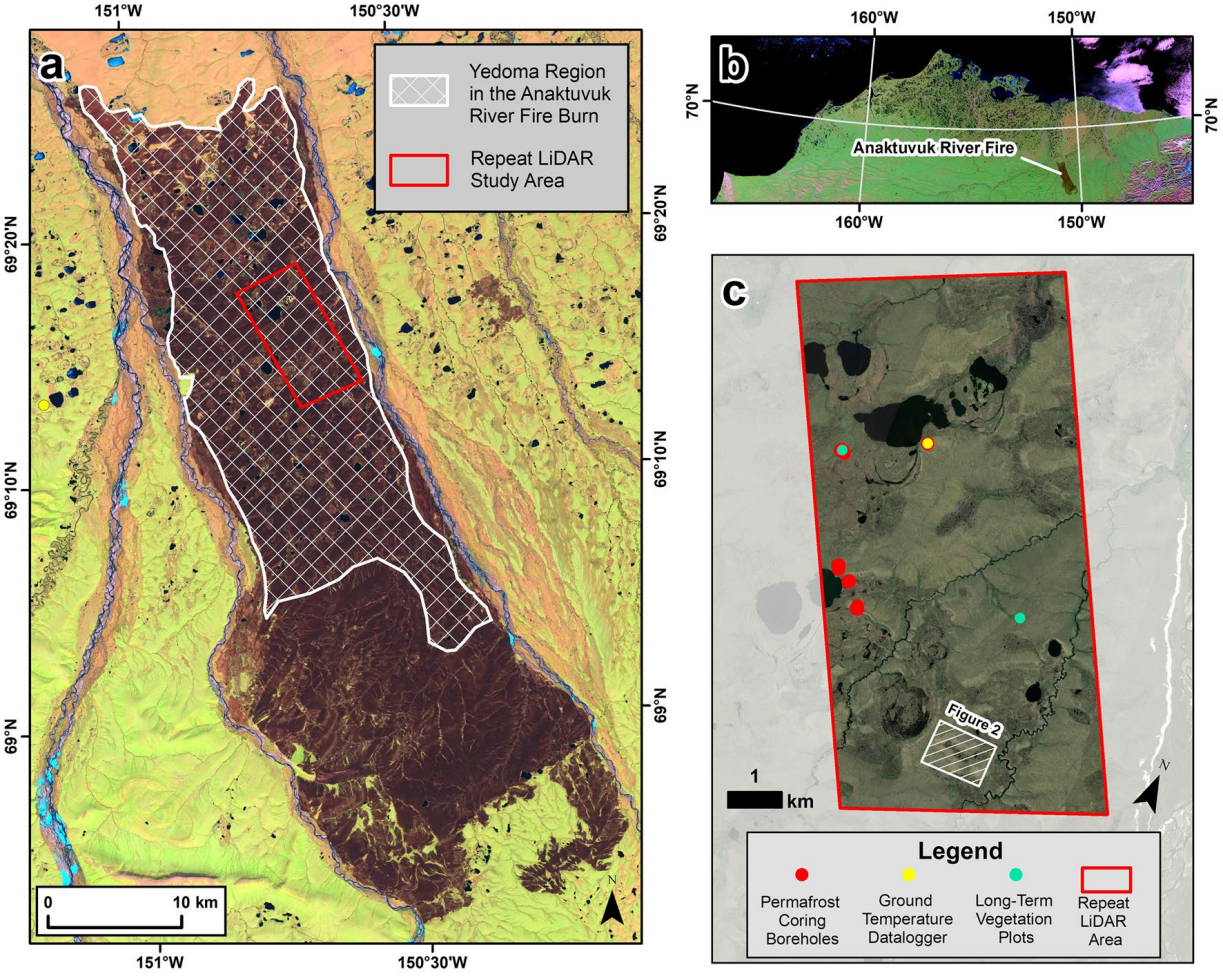
The Anaktuvuk River tundra fire study area. (**a**) Landsat-5 TM image acquired on 14 June 2008, the first summer after the Anaktuvuk River tundra fire, showing the  ~ 1000 km^2^ burn. The white cross-hatched polygon shows the extent of the Yedoma permafrost region in the burn area. The extent of the repeat LiDAR study area is outlined with the red box and the location of the ground temperature data logger in the unburned site is shown with the yellow dot. (**b**) Inset map showing a MODIS satellite image of northern Alaska and the location of the Anaktuvuk River fire burn area. (**c**) The 50 km^2^ repeat LiDAR study area showing the location of permafrost coring site, the ground temperature data logger, and the long-term vegetation plots from Jandt et al.^29^. The location of Fig. 2 is indicated with the white hatched rectangle. Map created in Esri® ArcMap™ 10.1.

The Anaktuvuk River tundra fire (Fig. 1a,b) burned more than 1000 km^2^ of permafrost-affected arctic tundra in northern Alaska in 2007. The fire is the largest historical recorded tundra fire on the North Slope of Alaska^2^. Fifty percent of the burn area is underlain by Yedoma permafrost (Fig. 1a) that is characterized by extremely high ground-ice content of organic-rich, silty soils and the occurrence of large, syngenetic polygonal ice wedges^36,37^. Given the extremely high ground-ice content, Yedoma is thought to be among the most vulnerable to fire-induced thermokarst in the Arctic. In our previous studies, we focused on investigating post-fire vegetation change^16,29^ and the impact of the Anaktuvuk River tundra fire on post-fire thermokarst development in the first seven years following the fire^26^. Results from our previous studies indicated that the role of tundra fires in initiating widespread thermokarst development in regions with ice-rich permafrost in the Arctic had been underestimated. However, due to a lack of previous long-term observations on the long-term effects of fire-induced permafrost thaw^24,27^, the trajectory of post-fire permafrost degradation in Arctic tundra remained uncertain.

In this study, we report observations on the evolution of near-surface permafrost in the Anaktuvuk River tundra fire burn area from 2009 to 2023 using repeat airborne LiDAR-derived elevation data, ground temperature measurements, and cryostratigraphic studies. Because the fire burned tundra vegetation and organic-rich soils underlain by a range of permafrost ground-ice conditions, long-term observations of the landscape evolution can help understand how different parts of the Arctic will change as fires become more common. Here we were interested in addressing two primary questions: (1) what is the trajectory and long-term effect of fire-induced permafrost degradation in the Arctic, and (2) can permafrost in burned tundra landscapes stabilize and recover under the current climate? To address these questions, we focus on a ~ 50 km^2^ mosaic of ice-rich permafrost terrain affected by the Anaktuvuk River tundra fire in the Yedoma ‘silt belt’ region that consists of Yedoma uplands, Yedoma slopes, and previously degraded Yedoma landforms, which include drained lake basins (DLBs) and stream channels.

## Results

### LiDAR change detection in terrain units

Between 2009 and 2014, permafrost thaw subsidence was detected across 15% of the 50 km^2^ terrestrial landscape area at a rate of 136.6 ha/yr (Table 1). The mean rate of detected subsidence was 0.08 m/yr and the total estimated volumetric land surface subsidence over this first time period (2009 to 2014) was 55.4 ha-m/yr. Between 2014 and 2021, following this initial period of post-fire thaw (2009 to 2014), subsidence was only detected across < 1% of the terrestrial landscape area at a rate of 5.2 ha/yr. During the latter period (2014 to 2021), the mean rate of detected subsidence was 0.05 m/yr and the total estimated volumetric land surface subsidence declined to 1.9 ha-m/yr, which corresponds to a decrease by a factor of ~ 30 (Table 1).

**Table 1:** Repeat LiDAR change detection and borehole coring results.

Terrain unit	Terrain unit area (ha)	Mean thaw subsidence at the landscape-scale^a^						Permafrost borehole coring results					
		2009 to 2014			2014 to 2021								
		Area (ha)	Area (% affected)	Volume (ha-m)^b^	Area (ha)	Area (% affected)	Volume (ha-m)^b^	Core location	Number of boreholes	Max. post-fire thaw depth (cm)^c^	Transient layer (cm)	Intermediate layer (cm)	Post-fire permafrost aggradation (cm)^d^
Yedoma uplands	2127	378	17.8	151	8	0.4	3	Trough	21	58.9	5.3	12.2	17.5
								Center	6	66.5	7.0	13.3	20.3
Yedoma slopes	321	131	40.8	62	10	3.2	4	Trough	11	49.8	2.8	3.6	6.5
								Center	1	52.0	8.0	0.0	8.0
DLBs and channels	2230	174	7.8	64	18	0.8	6	Trough	9	58.8	1.9	14.6	16.4
								Center	1	52.0	16.0	0.0	16.0
Totals	4678	683	14.6	277	36	0.8	13		49	57.5	4.6	10.3	15.0

Study area terrain units by landform showing portion with repeat airborne LiDAR from 2009, 2014, and 2021 and percent thaw subsidence in each over two time periods. We also summarize the borehole coring results for the 26 cores collected in June 2021, 21 cores collected in August 2022, and the 2 cores collected in August 2023 (*see Supplemental Information*), including information on the core location (ice wedge polygon center or trough), the number of boreholes per terrain unit and their location, maximum post-fire thaw depth, transient layer and intermediate layer thickness, and post-fire permafrost aggradation.

^a^Derived from multi-temporal airborne LiDAR change detected using the Geomorphic Change Detection software.

^b^Volume estimated by multiplying the detected area with thaw subsidence with the mean subsidence magnitude.

^c^Maximum post-fire thaw depth refers to the depth of the thaw unconformity at the time of the coring campaigns and does not account for thaw subsidence of the pre-fire surface. In the case of troughs, this depth refers to the depth to the top of the ice wedge at the time of the coring campaigns.

^d^Post-fire permafrost aggradation represents the thickness of the transient layer plus the intermediate layer.

**Figure 2:**
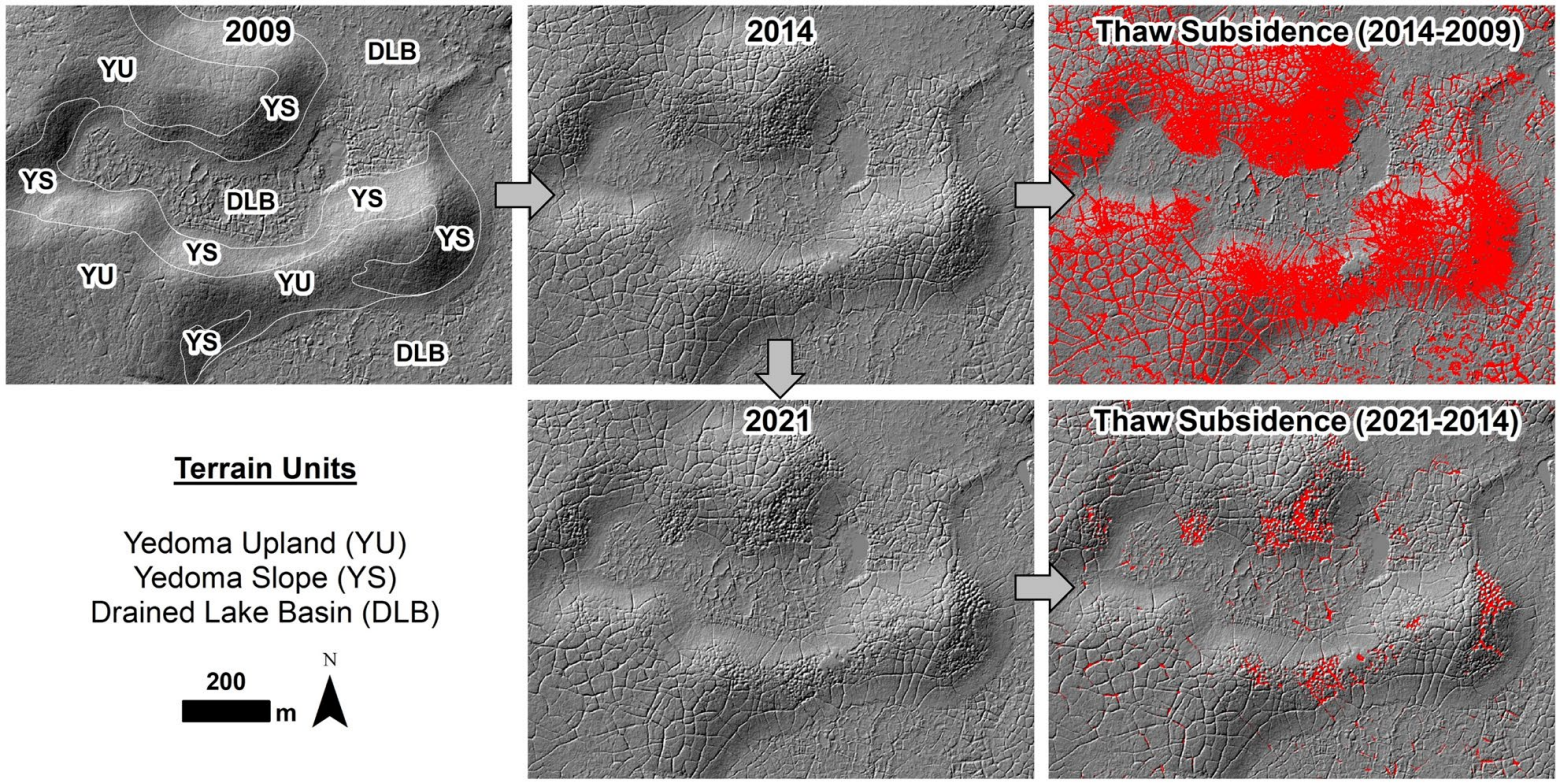
Documenting thaw subsidence changes in repeat LiDAR data. Hillshade images derived from the 1 m resolution LiDAR DTMs are shown from 2009 (upper left), 2014 (upper middle), and 2021 (lower middle). Thaw subsidence results in red were determined with the Geomorphic Change Detection software and shown for the period 2009 to 2014 (upper right) and 2014 to 2021 (lower right). Terrain unit symbols are shown in the upper left panel.

**Figure 3:**
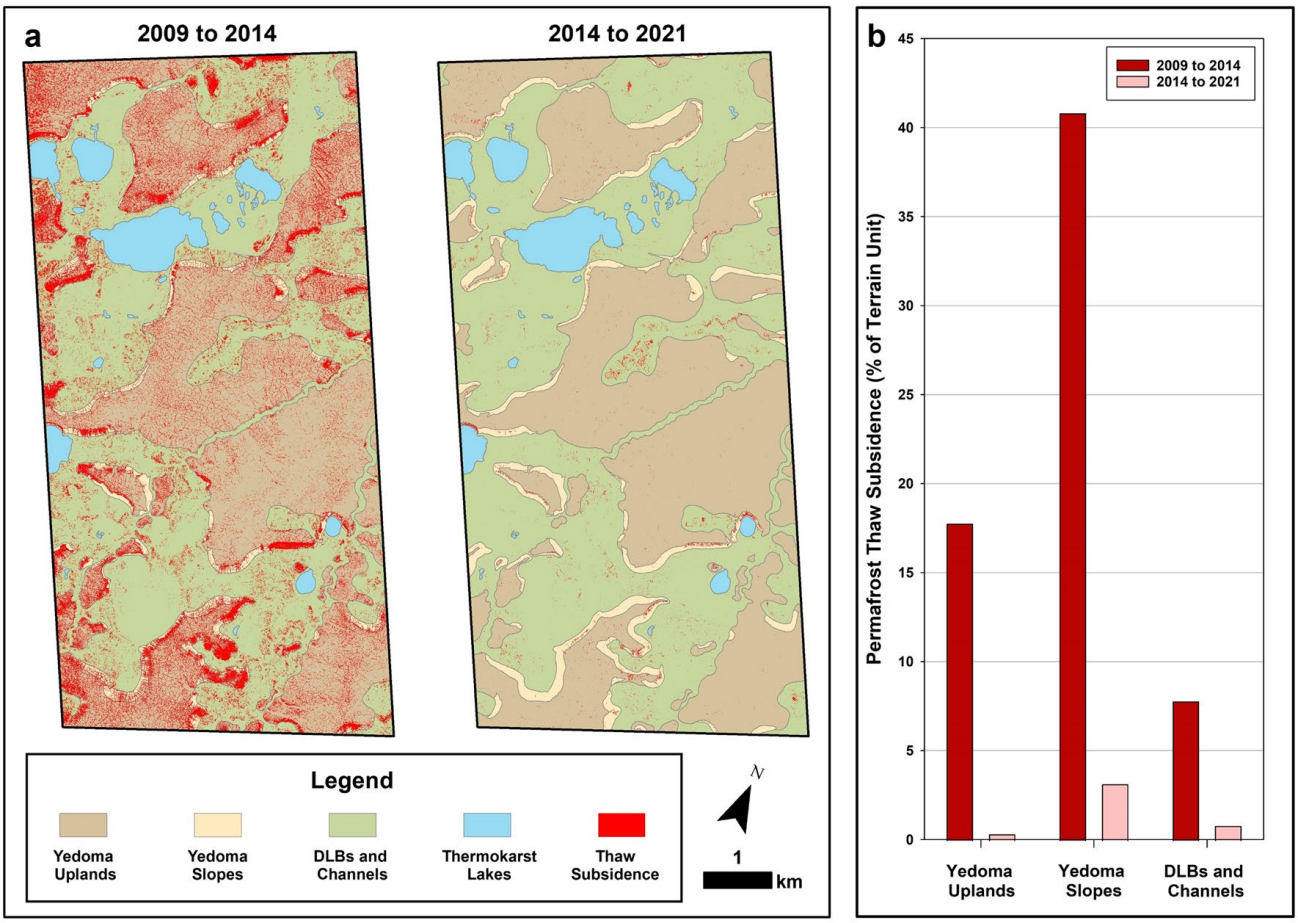
Post-fire stabilization of the thaw-affected permafrost landscape. (**a**) LiDAR change detection results for the time periods of 2009 to 2014 and 2014 to 2021 for the 50 km^2^ study area (see Fig. 1 for the location). The detected thaw subsidence is shown in red for both time periods and is overlain on the terrain unit map for the study area. Between 2009 and 2014, thaw subsidence was detected across 15% of the study area but between 2014 and 2021, additional thaw subsidence was detected in < 1% of the area, indicating stabilization of the thaw-affected permafrost terrain post-fire. (**b**) Permafrost thaw subsidence in both time periods according to terrain unit. Yedoma slopes and Yedoma uplands were most affected by permafrost thaw between 2009 and 2014. While thaw subsidence substantially declined across the entire study area, active thaw is still occurring along Yedoma slopes in localized locations.

The study area consists of three main terrain unit types—Yedoma uplands, Yedoma slopes, and DLBs (Fig. 2). Permafrost thaw subsidence varied among these terrain types and across the two time periods. Between 2009 and 2014, thaw subsidence was detected across 17.8% of the Yedoma upland area, 40.8% of the Yedoma upland slope area, and 7.8% of the DLB area (Fig. 3). Relative to the extent of varying terrain unit land area, Yedoma uplands contributed 8.1%, Yedoma slopes 2.8%, and DLBs 3.7% of the total detected thaw subsidence in the study area. This disparity was notable since Yedoma slopes only represented 7% of the study area relative to Yedoma uplands (43%) and DLBs (45%). Between 2014 and 2021, permafrost thaw subsidence magnitude decreased substantially with Yedoma uplands contributing 0.2%, Yedoma slopes 0.2%, and DLBs 0.4% (Table 1).

### Post-fire ground thermal regimes

Ground temperature measurements have been logged hourly since July 2009 at a burned and unburned Yedoma upland site with comparable pre-fire tundra and permafrost conditions. The continuous record of ground temperature measurements at a depth of 0.15 m and 1.00 m allows us to assess changes in mean annual ground temperatures (MAGT) as well as changes in the seasonal ground thermal regime characteristics in the active layer and permafrost, respectively (Table 2). In comparison with the unburned location, the MAGT in the burned location was 1.5 °C warmer in the active layer (0.15 m) and 1.4 °C warmer in the near-surface permafrost (1.00 m) as averaged between 2010 and 2022. Seasonally, the average relative warmth in the burn location at 0.15 m depth was greatest during the summer (2.7 °C), moderate in the winter (1.6 °C), and lowest in the spring (0.3 °C) and fall (0.3 °C) (Table 2). This relative warming at the burn location at 1.0 m depth was greatest in winter (3.2 °C), moderate in spring (0.8 °C) and fall (0.8 °C), and lowest in the summer (0.5 °C) (Table 2).

**Table 2:** Annual and seasonal ground temperature measurements and metrics from 2010 to 2022.

Metric	Location	Sensor depth (m)	Time period	Year												
				2010	2011	2012	2013	2014	2015	2016	2017	2018	2019	2020	2021	2022
Thawing degree days (TDDs)	Burned	0.15	Thaw season	567	578	615	578	511	607	555	493	358	496	353	316	324
	Unburned			192	177	250	267	165	208	234	203	163	267	173	222	249
Mean ground temperature (°C)	Burned	0.15	Winter	− 12.4	− 7.9	− 12.7	− 11.0	− 10.7	− 6.9	− 8.6	− 11.6	− 6.0	− 9.0	− 11.1	− 12.4	− 12.0
			Spring	− 0.3	− 2.3	− 0.9	− 1.6	− 0.6	− 1.3	− 1.8	− 0.5	− 1.4	− 1.0	− 0.8	− 1.2	− 0.7
			Summer	6.0	6.1	6.4	6.1	5.3	5.9	5.6	5.2	3.8	4.9	3.5	3.2	3.4
			Fall	− 0.3	− 2.3	− 0.9	− 1.6	− 0.6	− 1.3	− 1.8	− 0.5	− 1.4	− 1.0	− 0.8	− 1.2	− 0.7
			Annual	− 3.3	− 2.7	− 3.7	− 3.6	− 2.6	− 1.7	− 2.4	− 3.4	− 2.1	− 2.3	− 3.2	− 4.6	− 4.0
		1.00	Winter	− 7.2	− 3.9	− 7.6	− 5.9	− 5.8	− 3.4	− 5.1	− 7.3	− 3.3	− 5.9	− 6.6	− 7.7	− 8.4
			Spring	− 0.3	− 0.2	− 0.2	− 0.1	− 0.2	− 0.2	− 0.2	− 0.2	− 0.3	− 0.2	− 0.3	− 0.6	− 0.5
			Summer	− 1.0	− 0.7	− 0.7	− 0.7	− 0.6	− 0.4	− 0.6	− 0.9	− 0.8	− 0.7	− 0.9	− 1.2	− 1.1
			Fall	− 0.3	− 0.2	− 0.2	− 0.1	− 0.2	− 0.2	− 0.2	− 0.2	− 0.3	− 0.2	− 0.3	− 0.6	− 0.5
			Annual	− 4.0	− 2.8	− 4.1	− 3.7	− 3.2	− 2.3	− 3.0	− 4.0	− 2.4	− 3.0	− 3.6	− 4.6	− 4.4
	Unburned	0.15	Winter	− 9.6	− 10.6	− 13.3	− 13.0	− 15.0	− 9.9	− 10.6	− 12.3	− 9.4	− 9.2	− 12.6	− 16.0	− 12.1
			Spring	− 0.8	− 2.5	− 0.8	− 1.5	− 0.9	− 2.0	− 2.3	− 0.6	− 1.4	− 1.0	− 1.8	− 1.0	− 1.2
			Summer	2.1	1.9	2.7	2.9	1.8	2.2	2.5	2.2	1.8	2.9	1.8	2.4	2.7
			Fall	− 0.8	− 2.5	− 0.8	− 1.5	− 0.9	− 2.0	− 2.3	− 0.6	− 1.4	− 1.0	− 1.8	− 1.0	− 1.2
			Annual	− 3.9	− 4.7	− 5.2	− 5.2	− 5.0	− 4.0	− 4.1	− 4.5	− 3.8	− 3.1	− 4.8	− 5.8	− 4.7
		1.00	Winter	− 7.2	− 8.0	− 10.7	− 9.9	− 11.8	− 7.7	− 8.7	− 9.8	− 7.5	− 7.4	− 9.6	− 12.6	− 9.4
			Spring	− 0.7	− 1.7	− 0.7	− 1.0	− 0.9	− 1.4	− 1.4	− 0.7	− 1.1	− 0.6	− 1.2	− 1.0	− 0.9
			Summer	− 1.3	− 1.3	− 1.4	− 1.3	− 1.3	− 1.0	− 1.1	− 1.3	− 1.2	− 1.0	− 1.2	− 1.5	− 1.3
			Fall	− 0.7	− 1.7	− 0.7	− 1.0	− 0.9	− 1.4	− 1.4	− 0.7	− 1.1	− 0.6	− 1.2	− 1.0	− 0.9
			Annual	− 4.2	− 4.8	− 5.7	− 5.5	− 5.5	− 4.4	− 4.6	− 5.0	− 4.1	− 3.7	− 5.0	− 6.3	− 5.1

Ground temperature measurements have been logged hourly at two depths, 0.15 m and 1.00 m, at a burned and unburned Yedoma upland tundra location with comparable pre-fire tundra and permafrost conditions. The sensor at 0.15 m depth was used to calculate near-surface soil thawing degree days (TDDs) in the active layer at both the burned and unburned site. Ground temperate data at both depths have been aggregated to mean seasonal and mean annual ground temperatures from 2010 to 2022. Winter refers to December, January, and February; Spring refers to March, April, and May; Summer refers to June, July, and August; Fall refers to September, October, and November.

**Figure 4:**
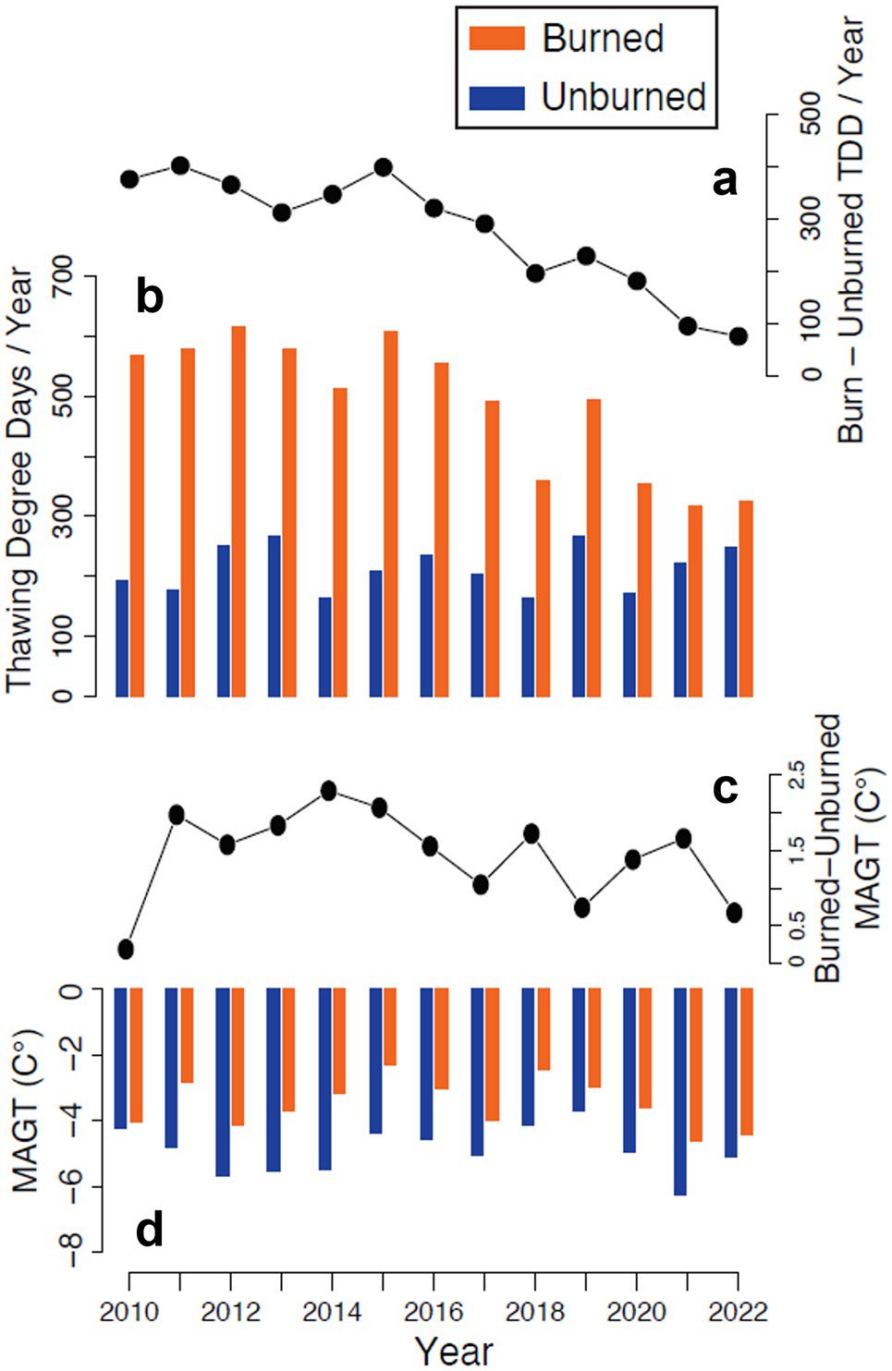
Post-fire, near-surface ground temperature changes. (**a**) The difference in near-surface soil (0.15 m depth) thawing degree days (TDDs) between burned and unburned tundra sites from 2010 to 2022. (**b**) The number of TDDs per year at a depth of 0.15 m for the burned and unburned tundra sites from 2010 to 2022. (**c**) The difference in mean annual ground temperature (1 m depth) between burned and unburned tundra sites from 2010 to 2022. (**d**) Mean annual ground temperature at 1 m depth for the burned and unburned tundra sites from 2010 to 2022.

Assessing annual variability in soil thawing degree days (TDDs) and MAGT differences between the burned and unburned sites shows an initial increase and then subsequent decrease in temperatures between 2010 and 2022 (Table 2). The number of TDDs of the active layer were > 3 × greater at the burn site (511) relative to the unburned control site (165) up to 7-years post-fire, but this relative warmth decreased to only 1.3 × greater by 2022 (Fig. 4 a,b). Warming of the permafrost initially lagged the temperature increases in the active layer as it deepened. The relative MAGT warmth in the burn location was + 0.2 °C for the 1-m depth three years after the fire, increased to 2.3 °C seven years after the fire, and then was reduced to 0.7 °C 15 years after the fire (2022) (Fig. 4c,d).

### Changes to near-surface permafrost

**Figure 5:**
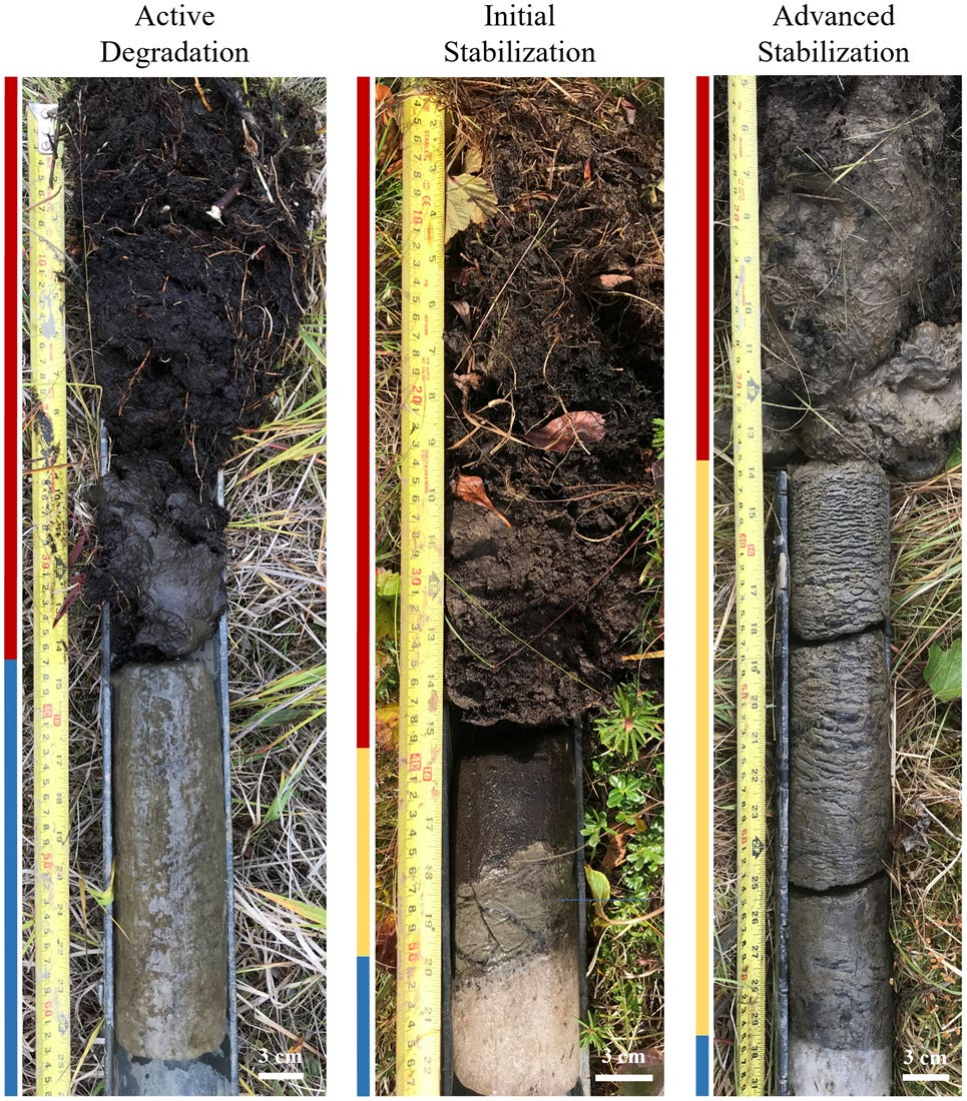
Active Degradation, Initial Stabilization, and Advanced Stabilization of ice wedges post-fire. Field photos taken of cores collected in ice wedge troughs in late August 2022 showing examples of an actively degrading ice wedge (left; *see Supplemental Information—AF26-22*), the initial stabilization of a degrading ice wedge (middle; *see Supplemental Information—AF13-22*), and an ice wedge with pronounced post-fire stabilization and permafrost aggradation (right; *see Supplemental Information—AF3-22*). Soil layers are indicated to the left of each core photo: thawed sediments (red), the transient and intermediate layer indicating post-fire aggradation of permafrost (yellow), and the unthawed wedge ice below (blue).

Permafrost boreholes drilled in ice wedge troughs and polygon centers in 2021–2023 (*see Supplemental Information*) revealed the presence of a thaw unconformity, a discontinuity in the nature and distribution of ground-ice bodies due to a past thaw event, that was overlain by a layer of frozen soil with distinctive cryostructures indicative of recovering permafrost^38^. This layer can be described as a transition zone in the upper permafrost, which consists of two layers: the transient and intermediate layers^38^. A transient layer is a result of interannual variability in the active‐layer thickness; it is defined as a layer of soil that belongs to the permafrost for several years and joins the active layer in the years with deeper seasonal thaw. An ice-rich intermediate layer forms below the transient layer due to a gradual decrease in the active‐layer thickness, mostly because of accumulation of organic matter. At our ice wedge sites, the combined thickness of the transient and intermediate layers above partially degraded wedges averaged 14.3 cm (n = 41), indicating aggradation of permafrost above the post-fire thaw unconformity (Fig. 5, Table 1). The average thickness of the recovering permafrost layer in ice wedge polygon centers and on top of baydzherakhs, residual thermokarst mounds formed due to ice wedge degradation on Yedoma slopes, was estimated to be 18.3 cm (n = 8), again indicating aggradation of permafrost following post-fire thermokarst development (Table 1).

Vulnerability of ice wedges to ongoing and future thermokarst, which is controlled by the thickness of the transient and intermediate layers, varies between different terrain units (Table 1, also *see Supplementary Information*). We targeted a range of apparent stages of ice wedge degradation and stabilization scenarios based on our previous research in northern Alaska^39^. Most of the ice wedges studied on the Yedoma upland are already recovering from fire-induced thermokarst. During our coring campaigns between 2021 and 2023, we could not find any actively degrading ice wedges on the Yedoma upland surface. The mean thickness of the combined transient and intermediate layers in ice wedge troughs, representing post-fire permafrost aggradation, was 17.5 cm and the average depth to wedge ice was 58.9 cm (n = 21). Ice wedges in the drained-lake basin were also protected relatively well. By 2022, the average post-fire permafrost aggradation was 16.4 cm, and average depth to wedge ice was 58.8 cm (n = 9). We encountered only one ice wedge that was degrading in August 2022. Post-fire stabilization of ice wedges has been slower on Yedoma slopes, where nearly half of the investigated ice wedges (5 of 11) were still experiencing ongoing degradation. Our coring assessments found that on average only 6.5 cm of post-fire permafrost aggradation had occurred above the non-degrading wedges (n = 6) and the average depth to wedge ice was 49.8 cm.

Ground-ice contents of the upper permafrost in general were very high (*see Supplemental Information*). Average excess-ice contents of frozen soils within the Yedoma upland surface and Yedoma upland slopes were 0.2% by volume for the frozen part of the active layer samples taken in June 2021 (n = 8), 9.4% for the transient layer (n = 9), 44.6% for the intermediate layer of the upper permafrost (n = 40), and 38.2% for the underlying syngenetic permafrost (n = 7). Average excess-ice contents of frozen soils within the drained-lake basin were 0.0% for the frozen part of the active layer (n = 2), 0.9% for the transient layer (n = 2), 42.1% for the intermediate layer of the upper permafrost (n = 3), 33.7% for the syngenetic permafrost (n = 2), and 26.2% for the para-syngenetic (refrozen talik deposits) permafrost (n = 1).

## Discussion

In the first ~ 15 years since the 2007 Anaktuvuk River tundra fire, the permafrost-influenced landscape appears to have evolved in three phases. The first phase (0 − 3 years post-fire) involved the increase in active-layer thickness and rapid development of active-layer detachment slides and thaw slumps on hillslopes^12,29,40^. However, these features remained limited spatially and stabilized relatively quickly. This initial stage of rapid permafrost degradation and stabilization was recognized by studies that concluded the fire had detectable, but limited impact on the permafrost-influenced terrain overall^12,20^. The second phase (3 − 7 years post-fire) involved widespread ice-wedge degradation and associated subsidence of ice-rich terrain in the Yedoma region^26^ due to further increases in active layer thickness that affected underlying ice-rich deposits (Fig. 1). The third phase (7 − 15 years post-fire) involves stabilization of partially degraded ice wedges and recovery of near-surface permafrost in the Yedoma region through the development of a transient and intermediate layer overlying a thaw unconformity. These observations help us to constrain the major shifts in landscape evolution following a severe tundra fire in our current climate where the mean annual air temperature in the region is  − 9 °C^41^.

The primary driver of widespread post-fire permafrost thaw was the consumption of both the tundra vegetation and the insulating soil surface organic material through combustion. Post-fire changes in albedo^42,43^ and the loss of the insulating soil organic layer resulted in warming near-surface soil temperatures in the summer^44,45^. This is reflected in the relatively high near-surface soil TDDs in the burned locations during the first several years post-fire that led to an increase in active-layer thickness. Coinciding with this period of active-layer thickness increases was also the initial rapid, recovery of the *Eriophorum vaginatum* tussocks^12,29^. The quick recovery and vigorous regrowth of tussocks, from 6 to 60% between 2008 and 2011^29^, as well as the post-fire influx of tall graminoid species^16^, likely contributed to an increase in wind-blown snow accumulation in the winter that effectively trapped the summer warmth in the ground, contributing to the preferential warming of the permafrost during the fall, winter, and spring. Combined, this led to thaw of ice-rich, near-surface permafrost, including the pre-fire, ice-rich intermediate layer above ice wedges, prompting thermokarst development. The cryostratigraphy of a permafrost core collected adjacent to the location of the ground temperature data logger (Fig. 1c) indicates a thaw unconformity at a depth of 65 cm (active-layer thickness in 2023 was 52 cm) that likely coincided with the peak warming of the near-surface ground temperatures measured up to 2015.

The combination of post-fire vegetation and soil surface organic layer recovery has played a crucial role in the stabilization of the permafrost terrain and post-fire aggradation of permafrost. Jandt et al.^29^ showed that in the first ten years following the Anaktuvuk Fire *Eriophorum vaginatum* tussocks and species of willow shrubs were growing more vigorously in the burned tundra area relative to unburned tundra, that deciduous shrub cover had recovered to pre-fire levels, and that 5 cm of moss and plant litter had been added to the soil surface layers. The changes in the post-fire tundra ecosystem, in particular development of surface soil organic layers, has likely contributed to a cooling of ground temperatures in the active layer and permafrost. Due to the decrease in the active layer thickness, on average, 15 cm of permafrost has aggraded (Table 1). Chen et al.^13^ similarly found that several decades following tundra fires in northwestern Alaska that the shrub growth was enhanced in upland terrain but reduced in lowland terrain as the two landscape settings responded differently to post-fire thermokarst. The thermal effects of a slower and partial post-fire recovery of vegetation and soil surface organic layers in lowlands and a faster and more complete post-fire recovery in uplands can likely explain why muted but ongoing thermokarst continues in lowlands and especially Yedoma slopes, and why thermokarst has mostly ceased on Yedoma uplands.

The permafrost temperature data indicate that even though ground cooling has occurred since 2015, the MAGT is still warmer than the adjacent unburned control site. The increase in accumulated wind-blown snow in the fire area due to increased microtopography, resulting from post-fire ice wedge melting that caused differential thaw settlement, and the taller stature plants (such as willows, dwarf birch, and graminoids) on the stabilizing upland Yedoma surfaces are likely partially responsible for the warmer mean annual ground temperatures in the burn area relative to the control in 2018, 2020, and 2021, despite general cooling of the permafrost since 2015. More snow accumulation arising from increased microtopography contributed to higher minimum wintertime ground temperatures of ~ 6 °C at 1 m depth elsewhere in Alaska^46^. In the future, it would be good to study how this increased snowpack on the Yedoma upland surfaces might be affecting the ongoing degradation of ice wedges on Yedoma slopes and lowland terrain as well as the expansion rate of thermokarst lakes and gully formation.

Paired field and remote sensing-based observations of the sustained impact of tundra fires on permafrost are rare^16,17,27^. Exploiting detailed field data collection efforts with the ability to scale-up observations using remote sensing data is needed to more holistically document the short- and long-term effects of tundra fires on permafrost terrain^14–16,47^. Our findings show that the current climate conditions and ecosystem properties have contributed to the development of a recovered transient and intermediate layer as permafrost aggrades in the first ~ 15 years following the fire-induced degradation (Figs. 5).

**Figure 6:**
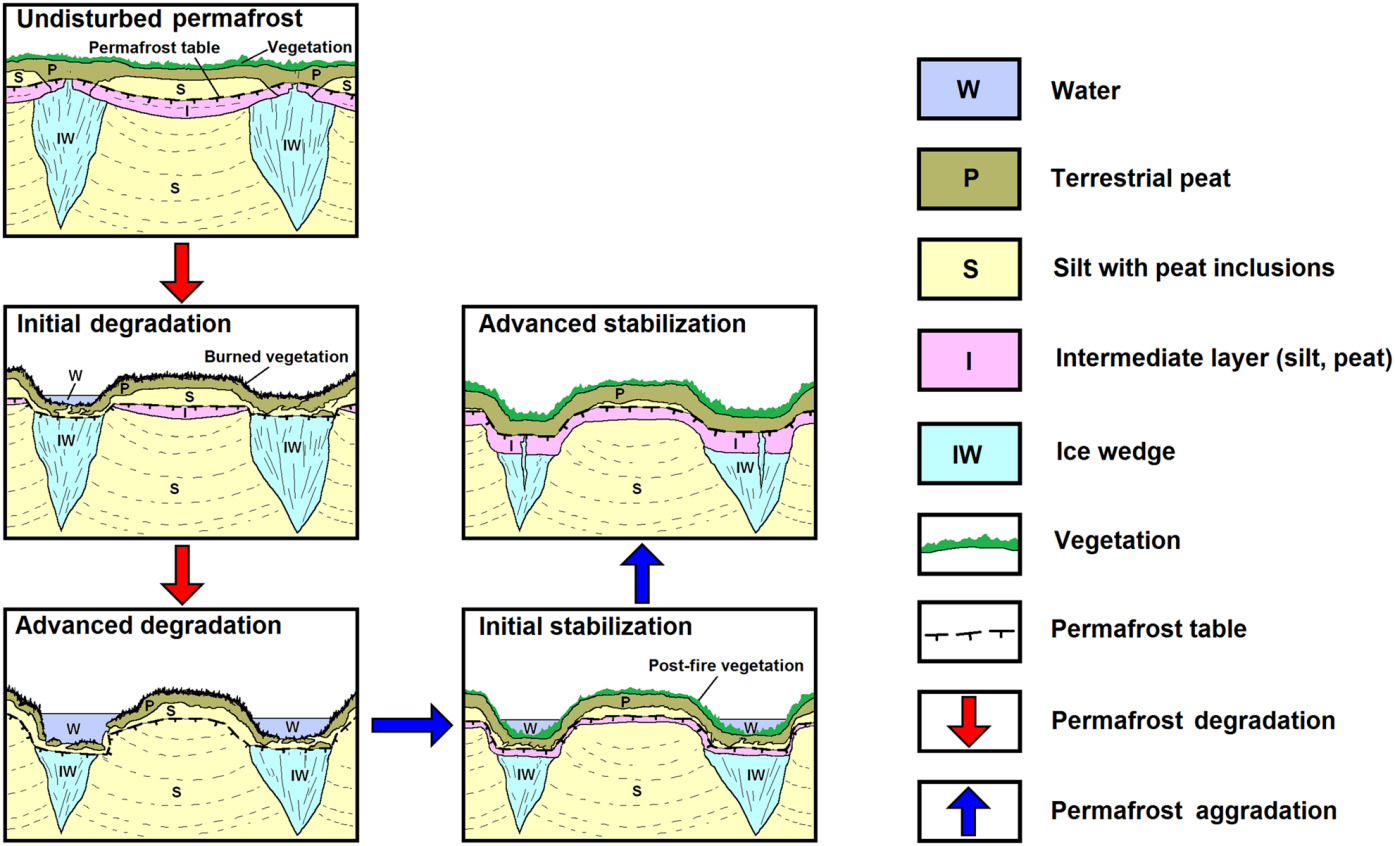
Conceptual diagram of post-fire permafrost degradation, stabilization, and aggradation in northern Alaska. At the Anaktuvuk River tundra fire site, post-fire degradation of the upper permafrost, which affected both ice wedges and central parts of ice wedge polygons, waned after 2014. Accumulation of organic matter (especially growth of aquatic vegetation in ice-wedge troughs) and aggradation of permafrost have increased the depth from the ground surface to ice wedges. The development of an ice-rich intermediate layer provides long-term stability for ice wedges and near-surface permafrost, building resilience to future thaw in a warming Arctic.

The conceptual diagram shown in Fig. 6 illustrates the process of post-fire permafrost degradation and stabilization in northern Alaska. Following a tundra fire, degradation of the upper permafrost, which affects both ice wedges and the central parts of ice-wedge polygons, initially intensifies. Increase in the active-layer thickness during the initial degradation stage leads to partial thawing of ice wedges with formation of shallow troughs. Water impoundment and additional snow accumulation in troughs leads to further melting of ice wedges and deepening of troughs (advanced degradation stage). However, this process slows after several years and then reverses due to the regrowth of vegetation and the accumulation of organic matter, which lead to significant decrease in the active-layer thickness. As a result, the lower part of the active layer transforms into perennially-frozen state forming transient and intermediate layers. Development of the ice-rich intermediate layer effectively increases the distance between the ground surface and underlying ice-rich permafrost and/or ice wedges. These processes contribute to the formation of a protective barrier, enhancing the long-term stability of permafrost. Consequently, this increases the resilience of the near-surface permafrost against future thaw events.

Our findings are consistent with Chen et al.^24^ that showed that more than 50% of the thermokarst development occurred in the first decade following tundra fires in northern Alaska, after which thermokarst processes declined rapidly. Similarly, Miller et al.^27^ highlighted the resilience of permafrost in an old burn near the Chandler River in northern Alaska, where the density of ice wedge thermokarst pits inside the tundra fire disturbed area decreased from 1948 to 2017; whereas the density of pits increased in the adjacent unburned tundra area as a result of climate change over that time period.

The Yedoma region of the Anaktuvuk River tundra fire shows evidence of being disturbed by fire-induced permafrost thaw. The widespread increase in microtopography due to ice wedge thermokarst will likely remain apparent for at least several decades, if not longer^16,27^. However, due to post-fire vegetation changes, soil reaccumulation, and the development of an ice-rich intermediate layer that provides long-term stability for ice wedges, much of the burned and thaw-affected Yedoma region is now underlain by a permafrost type that is likely more resilient to future thaw in a warming Arctic^27,39,48^. Our previous observations in northern Alaska showed that the intermediate layer on top of recently degraded ice wedges is usually ∼2–3 times thicker than the intermediate layer above undegraded wedges and, therefore, these partially degraded and stabilized ice wedges become much less vulnerable to future thermokarst^39,48–50^. As a result, the perturbation needed to trigger subsequent subsidence in this post-fire terrain is now higher than it would have been in the absence of post-fire thermokarst and subsequent permafrost recovery.

Estimates from the large Anaktuvuk River tundra fire of 2007 suggest that vegetation and combustion of soil organic carbon during the fire emitted an amount of carbon equivalent to the annual net C sink for the entire Arctic biome^22^. Permafrost degradation and thermokarst development in the first seven years following the fire^26^ have also likely led to the mobilization of carbon and release of nutrients previously frozen in permafrost^18,34,51,52^. However, the results presented in this study indicate that in the Yedoma region, thaw was primarily limited to the upper ~ 1–2 m of the surficial deposits that are primarily of Holocene age^37,53^. As described above, it is likely that older permafrost carbon at depth in the Anaktuvuk River fire burn area is now more thoroughly protected from future thaw-induced disturbances. Taken together, our observations highlight that the initial degradation of ice-rich permafrost terrain in the first ten years following the Anaktuvuk River tundra fire has been followed by a period of permafrost aggradation and terrain stabilization.

## Methods

The data that support the findings of this study are published at the Arctic Data Center^54^ and described in more detail below.

### Repeat LiDAR analysis

We analyzed summertime airborne LiDAR data from 2009, 2014, and 2021 in a 50 km^2^ area to quantify permafrost thaw subsidence in the aftermath of the Anaktuvuk River tundra fire. The 2009 LiDAR data was acquired with an Optech ALTM 3100 LiDAR system flying at an altitude of 1000 m, the 2014 LiDAR data was acquired with a Riegl VQ 480i LiDAR system flying at an altitude of 600 m, and the 2021 LiDAR data was acquired with a Reigl VQ-580ii LiDAR system flying at an altitude of 750 m. The estimated point density of the LiDAR datasets was 2, 8, and 16 points per square meter (ppm). All datasets were processed into 1 m spatial resolution gridded raster digital terrain models (DTMs) using the software Quick Terrain Modeler (QTM) v. 8.2. The vertical accuracy of the datasets was assessed to be 0.13, 0.10, and 0.10 m, respectively, based on field survey differential GPS data. All LiDAR datasets were processed as NAD83, UTM zone 5N using Ellipsoid Heights in meters.

Permafrost thaw subsidence was determined using the Geomorphic Change Detection (v. 7) stand-alone software package^55,56^. Thaw subsidence was determined based on a per-pixel basis in the orthogonal and concurrent DTMs using default values for assessing vertical change in airborne LiDAR data in the GCD software. This entailed creating a raster error surface grid using 0.15 m and quantifying detectable change using the default confidence level (0.80). The result was a differential DTM (dDTM) identifying changes greater than 0.24 m of subsidence between each of the airborne LiDAR datasets. Areas identified as representing thaw subsidence due to noise in the LiDAR data caused by surface water features were manually removed from further analysis. We also removed changes associated with thermokarst lake margins and their outlets as we were interested in assessing thaw subsidence associated with top down permafrost thaw and thermokarst development in ice wedge polygonal terrain. We also did not assess increases in elevation in the datasets due to potential noise introduced by post-fire regrowth of low-stature, dense tundra vegetation. Areas identified as representing permafrost thaw subsidence were then aggregated according to terrain units that represent (1) Yedoma uplands, (2) Yedoma slopes, and (3) drained lake basin lowlands and stream valleys.

### Terrain unit mapping

The Land Facet Corridor Designer extension for ArcGIS^57^ was used to develop a terrain unit map for the study area based on calculation of a topographic position index (TPI) at two different scales as described in Jones et al.^26^. The terrain unit map was modified in this study to further distinguish Yedoma slopes from Yedoma uplands. This was accomplished through creation of 30 m resolution DTM from the 2009 LiDAR data to identify slopes > 3°. These areas were manually digitized and incorporated into the terrain unit map as an additional class. The final set of terrain units appearing in the study area are (1) Yedoma uplands, (2) Yedoma slopes, and (3) drained lake basin lowlands and stream channels. These three classes were used to analyze variability in subsidence across the study domain between 2009 and 2014 and between 2014 and 2021.

### Ground temperature measurements

An ONSET HOBO U23-002 datalogger with two external thermistors was installed in a 5 cm diameter borehole drilled to a depth of 1.00 m below ground level in July 2009. The location of both logger installations targeted Yedoma upland surfaces with tussock tundra. The dataloggers were configured to log ground temperature at hourly intervals at depths of 0.15 m (active layer) and 1.00 m (permafrost). The ground temperature data were aggregated into daily mean values and then summarized as mean seasonal and annual ground temperatures. Accumulated soil thawing degree days (TDDs) were calculated for the active layer sensor. The data were plotted as the difference in TDDs and ground temperature between the burned and unburned tundra locations to assess the effect of the fire on active layer and permafrost temperatures, respectively, between 2010 and 2022.

### Permafrost borehole coring campaigns

Between 2021 and 2023, we conducted three permafrost coring campaigns in the Yedoma region of the Anaktuvuk River tundra fire. In June 2021, we performed field work in the area affected by the 2007 Anaktuvuk River fire to study cryostratigraphy and ground-ice content of the upper permafrost and to compare permafrost conditions in burned and unburned areas (Fig. 1c). This campaign focused on coring the same locations as 2010^58^ as well as sites near a long-term vegetation transect (B37) location^29^. During June 2021, 21 boreholes were drilled in the centers of ice-wedge polygons and ice-wedge troughs within five study sites in the burn area; four of them were located within Yedoma uplands and/or Yedoma slopes, and one—within a drained-lake basin. Gravimetric and volumetric moisture contents and excess ground-ice content were measured on frozen samples obtained from these cores (*see Supplementary Information*). In August 2022, we revisited the 2021 coring sites and checked the status of the same ice wedges at the end of thawing season. During this study, 21 boreholes were drilled in ice-wedge troughs in the burn area (*see Supplementary Information*). In August 2023, we drilled two boreholes (an ice wedge polygon center and an ice-wedge trough) adjacent to the near-surface ground temperature monitoring station located inside the burn area. All permafrost cores were collected with a SIPRE core barrel with a diameter of 7.5 cm. We analyzed the permafrost cores to describe near-surface permafrost cryostratigraphy; evaluate ice content of the active layer (if still frozen), transient layer, and intermediate layer of the upper permafrost; and assess post-fire permafrost aggradation.

### Supplementary Information

Supplementary Information.

## Data Availability

The data that support the findings of this study are published at the Arctic Data Center^54^.
