# Supplementary material for: Post-fire stabilization of thaw-affected permafrost terrain in northern Alaska
Source: Sci Rep. 2024 Apr 11;14:8499. doi: 10.1038/s41598-024-58998-5 (PMC11009396; doi:10.1038/s41598-024-58998-5)
Supplement: Supplementary file 1 — Supplementary Information. [file 41598_2024_58998_MOESM1_ESM.pdf]

Permafrost boreholes, 2007 Anaktuvuk River Fire study area (September 2010, June 2021, August 2022, and August 2023)

In September 2010, we performed field work in the area affected by the 2007 Anaktuvuk River Fire. (**Figures S1 and S2, Table S1**). Our research included studies of cryostratigraphy and ground-ice content of the upper permafrost. During this study, eight boreholes were drilled mainly within ice-wedge polygon centers, and gravimetric moisture contents were measured (**Table S1**).

In June 2021, we performed field work in the same area. Our research included studies of cryostratigraphy and ground-ice content in the area affected by the 2007 Fire; we also compared permafrost conditions in burned and unburned areas. During this study, 31 boreholes were drilled within five study sites, four of them were located within yedoma uplands and/or yedoma slopes, and one – within a drained-lake basin (**Figure S1, Table S2**). Some of these boreholes were drilled at the same locations where we had already performed drilling in September 2010. Total length of obtained cores was 29.2 m, and 71 samples were collected; gravimetric and volumetric moisture contents and excess ground-ice content were measured (**Table S2**). Entire cores were described and photographed in the field (examples of photographs are shown in **Figures S3 to S6**).

In August 2022, we revisited the 2021 coring sites and checked the status of the same ice wedges at the end of thawing season (**Table S2**). During this study, 21 boreholes were drilled in ice-wedge troughs, and four samples were collected; gravimetric and volumetric moisture contents and excess ground-ice content were measured (**Table S2**). Entire cores were described and photographed in the field (examples of photographs are shown in **Figures S7 to S9**).

In August 2023, we continued our previous studies and drilled two boreholes (in the center of an ice-wedge polygon and in an ice-wedge trough) with a SIPRE corer at one additional site (ARF23) (**Table S2**). The cores were described, photographed, and sampled in the field. Examples of photographs of frozen soils are shown in **Figures S10 to S11**.

Average excess-ice contents of frozen soils (2021 coring data) within yedoma uplands and yedoma slopes were 0.15% by volume for the frozen part of the active layer (n=8), 9.37% for the transient layer (n=9), 44.61% for the intermediate layer of the upper permafrost (n=40), and 38.17% for the syngenetic permafrost (n=7). Average excess-ice contents of frozen soils (2021 coring data) within drained-lake basin were 0.00% for the frozen part of the active layer (n=2), 0.86% for the transient layer (n=2), 42.13% for the intermediate layer of the upper permafrost (n=3), 33.67% for the syngenetic permafrost (n=2), and 26.18% for the para-syngenetic (refrozen talik) permafrost (n=1). Based on the 2022 and 2023 coring data, average excess-ice contents of the intermediate layer within yedoma were 29.38% (n=4, 2022) and 43.99 (n=5, 2023).

Permafrost boreholes drilled in ice-wedge troughs in 2022 and 2023 (15-16 years post-fire) revealed the presence of a thaw unconformity that in most cases was overlain by a recovered permafrost layer (includes the transient and intermediate layers above partially degraded ice wedges) that averaged 14.2 cm (n=20, August 2022 and August 2023 boreholes), indicating aggradation of permafrost following

post-fire thermokarst development. The average thickness of the recovered permafrost layer in polygon centers and on top of baydzherakhs was estimated to be 18.3 cm (n=8, June 2021 and August 2023 boreholes). For the polygon centers and ice-wedge troughs combined, the average thickness of recovered permafrost layer was 15.4 cm (n=28, centers June 2021 and August 2023, n=8; troughs August 2022 and August 2023, n=20).

Nine of the 20 ice-wedge troughs were dry, and water depth in 11 flooded troughs varied from 2 to 30 cm, 13.8 cm average (August 2022 and August 2023 data). The depth to wedge ice in the troughs varied from 34 to 84 cm (57.2 cm average, n=20). Four ice wedges of 20 were still degrading, and two more ice wedges were vulnerable (protective frozen soil layer above wedge ice <10 cm). The thickness of the recovered permafrost layer varied from 0 to 40 cm (14.2 cm average). Our data show that there is no correlation between water depth in ice-wedge troughs and thickness of the frozen protective layer (post-fire transient and intermediate layers combined) (**Figure S12**) and water depth and depth to ice wedges (**Figure S13**).

Vulnerability of ice wedges to thermokarst, which is controlled by the thickness of the frozen protective soil layers, varies between different terrain units (yedoma uplands, yedoma slopes, and drained-lake basins):

- Most of ice wedges within **yedoma uplands** have recovered from fire-induced thermokarst: in 2022 and 2023, we could not find any degrading ice wedges, the average thickness of frozen protective soil layer was 18.6 cm (n=11), and average depth to wedge ice was 60.5 cm (n=11). Only two of 11 ice wedges were still vulnerable (with protective layers 3 and 6 cm thick), while all other wedges were protected by frozen soil layers 12 to 40 cm thick.
- Ice wedges in the **drained-lake basin** were also protected relatively well: in August 2022, the average thickness of frozen protective soil layer was 12.8 cm (n=4), and average depth to wedge ice was 57.3 cm (n=4). Only one ice wedge was degrading in August 2022 (it was in the elevated part of the trough, which did not degrade immediately after the fire but started degrading more recently), while the other three wedges were well protected by frozen soil layers 14 to 20 cm thick.
- Post-fire stabilization of ice wedges has been much slower within **yedoma slopes**, where three of the five ice wedges were still experiencing degradation. In August 2022, the average thickness of frozen protective soil layer was 5.6 cm (n=5), and average depth to wedge ice was 49.8 cm (n=5). Two ice wedges, which were not degrading in 2022, were relatively well protected by frozen soil layers 12 and 16 cm thick.

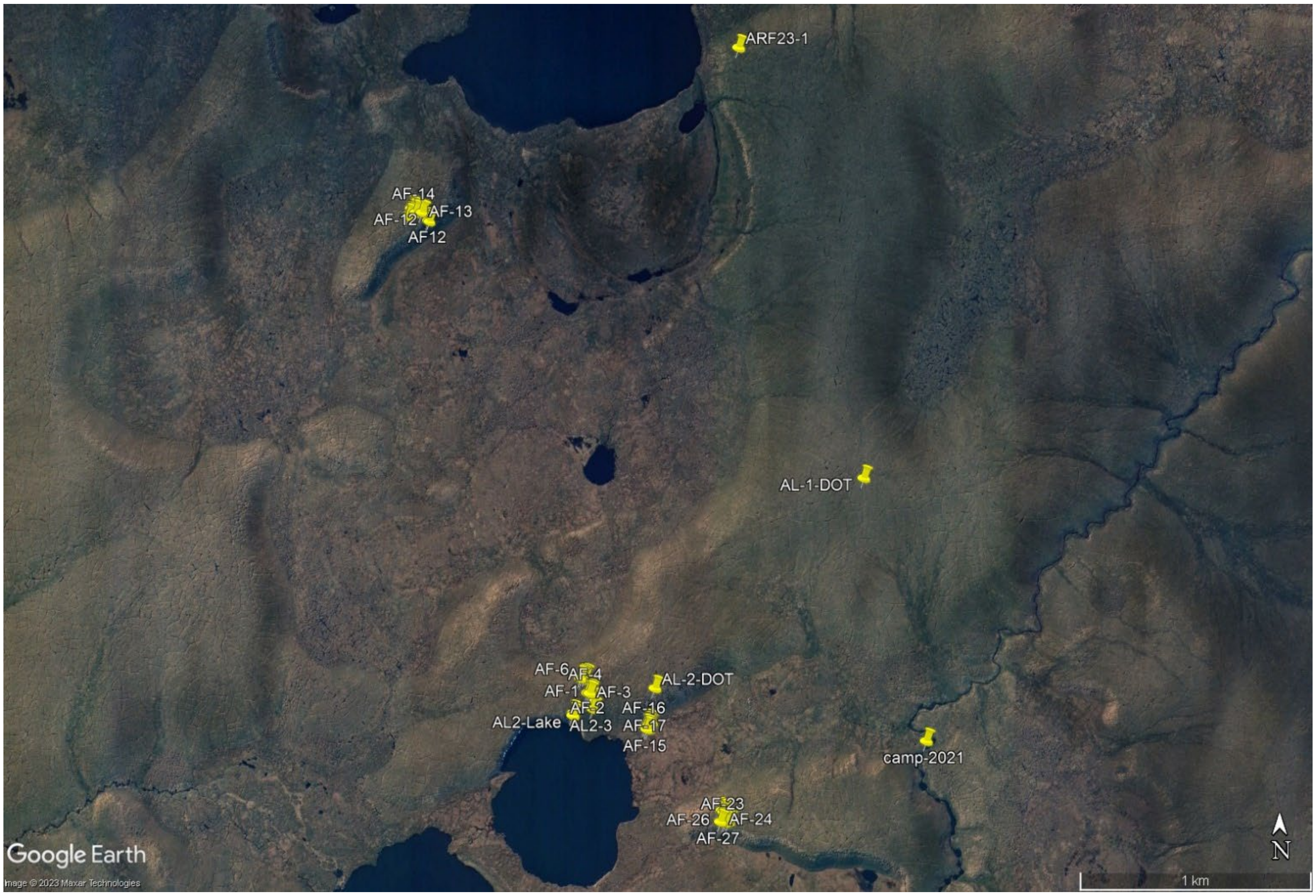

Figure S1. Location of boreholes drilled in September 2010, June 2021, August 2022, and August 2023. Location of the ARFU study site (June 2021; approximately 19 km west of the 2021 campsite) is not shown.

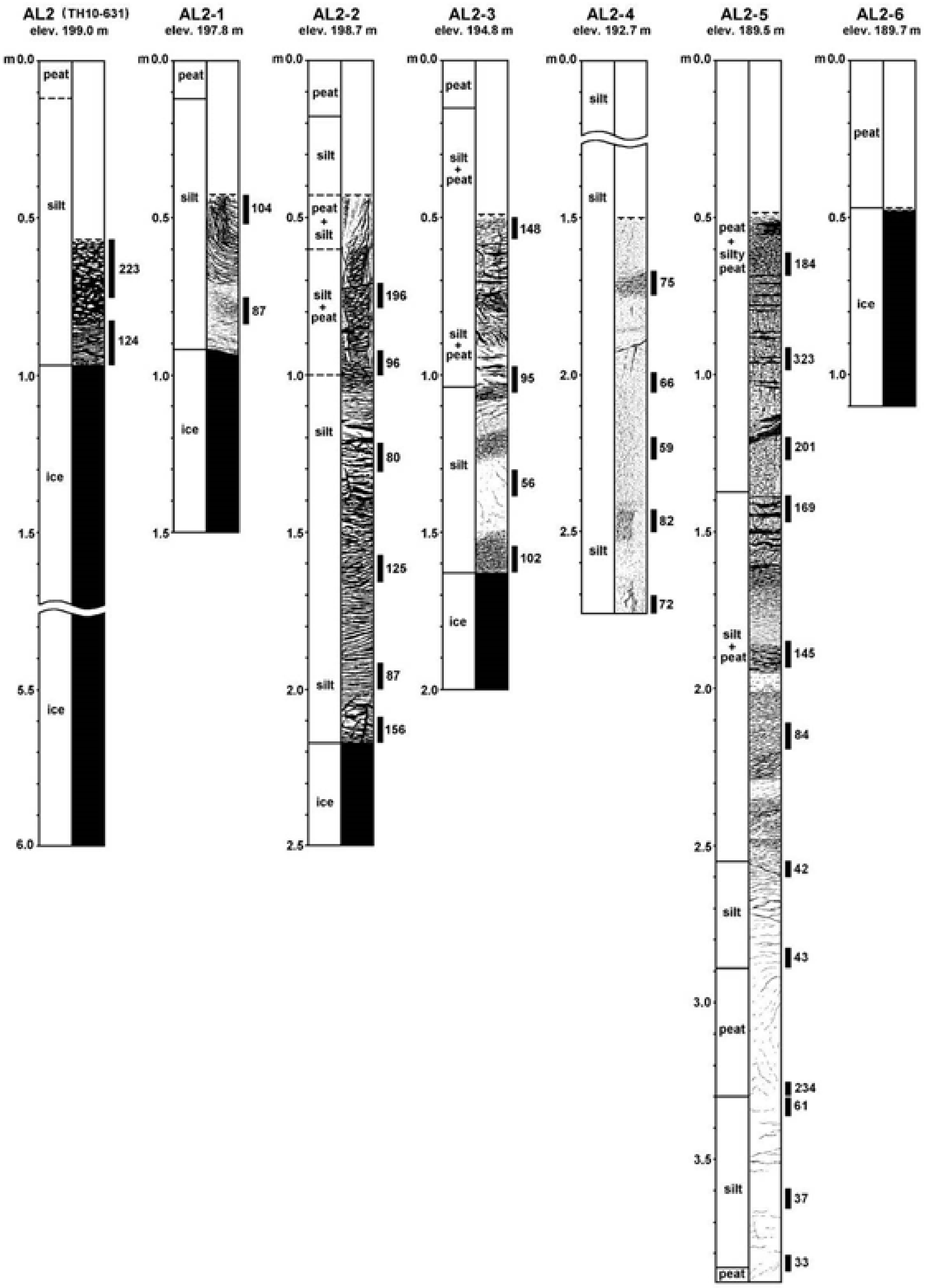

Figure S2. Cryostratigraphy of the upper permafrost and gravimetric moisture contents, Anaktuvuk River Fire study area, September 2010.

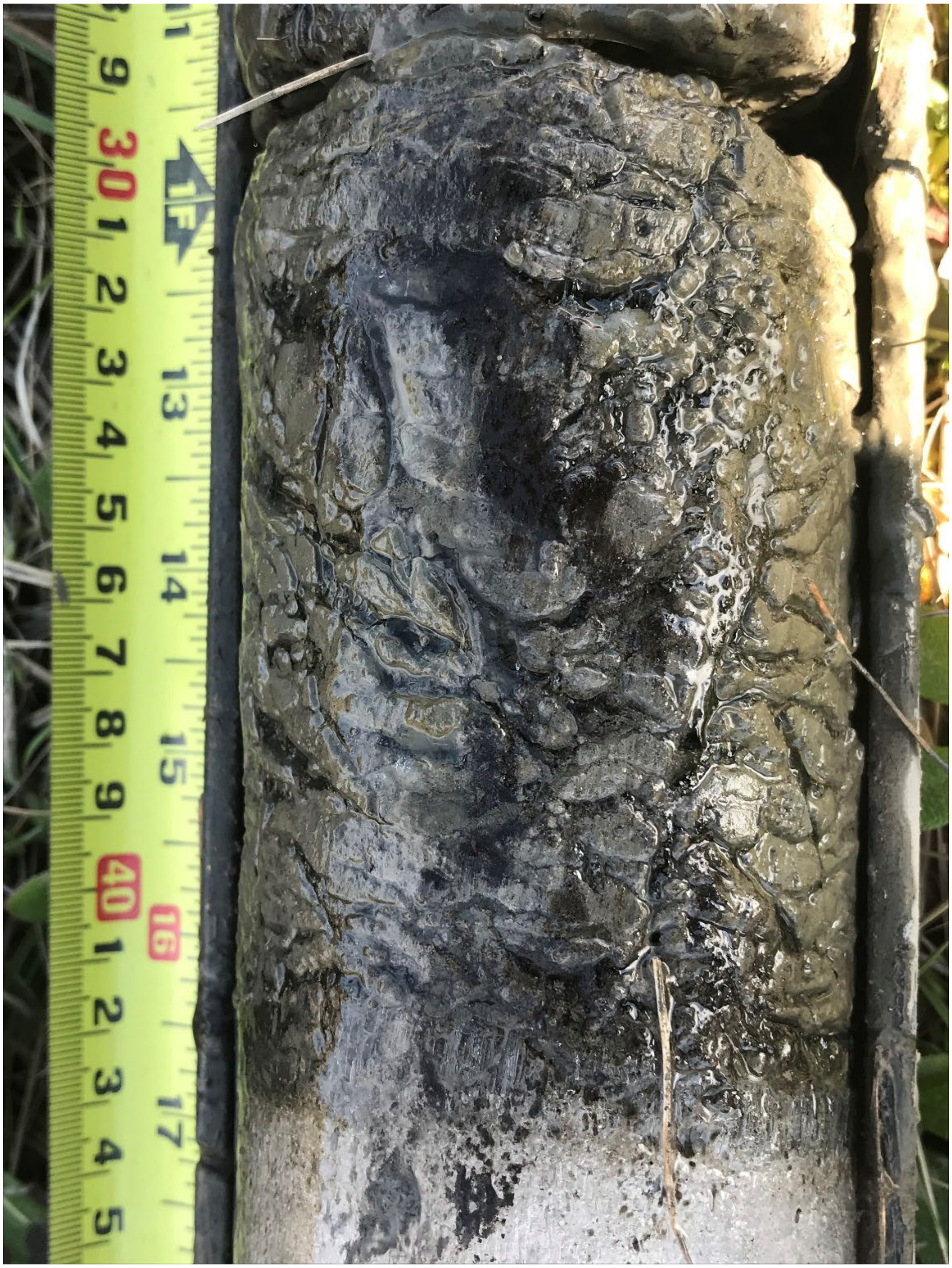

Figure S3. Ice-rich intermediate layer (33-41 cm) above wedge ice, silt with peat inclusions, excess ice content 30.6% (33-39 cm); borehole AF-3, June 24, 2021; this borehole was drilled in the 40-cm-deep trough, water depth 13 cm. Occurrence of this layer indicates post-fire stabilization of partially degraded ice wedge.

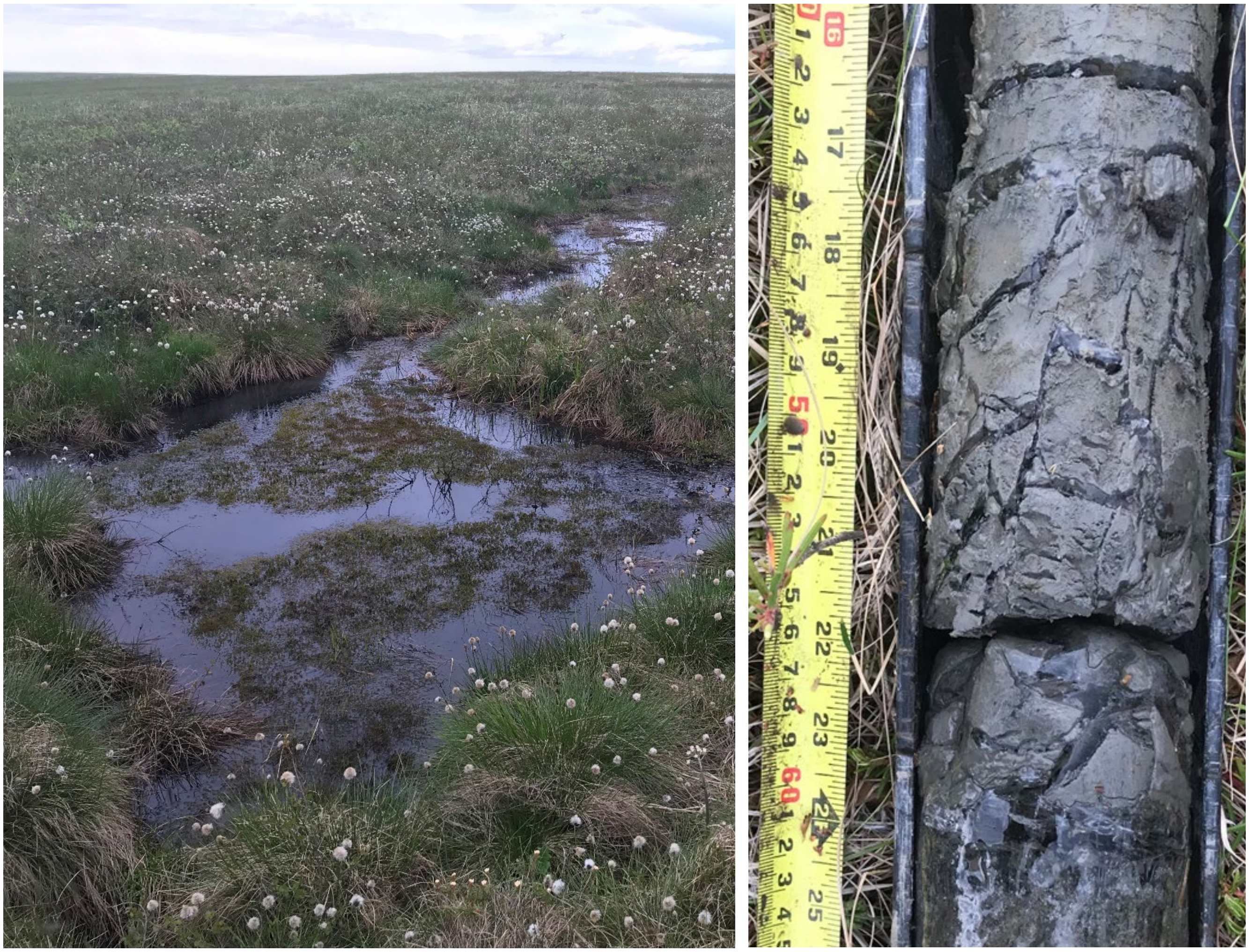

Figure S4. Borehole AF-6, June 24, 2021; this borehole was drilled in the 40-cm-deep trough, water depth (including aquatic moss) 21 cm.

Left: Borehole location. Thermokarst pond in the ice-wedge trough. Accumulation of aquatic mass resulted in decrease of the active-layer thickness and formation of the intermediate layer.

Right: Ice-rich intermediate layer (46-60 cm) above wedge ice, silt, excess ice contents 24.0% (46-52 cm), 50.7% (56-60 cm); Occurrence of this layer indicates post-fire stabilization of partially degraded ice wedge.

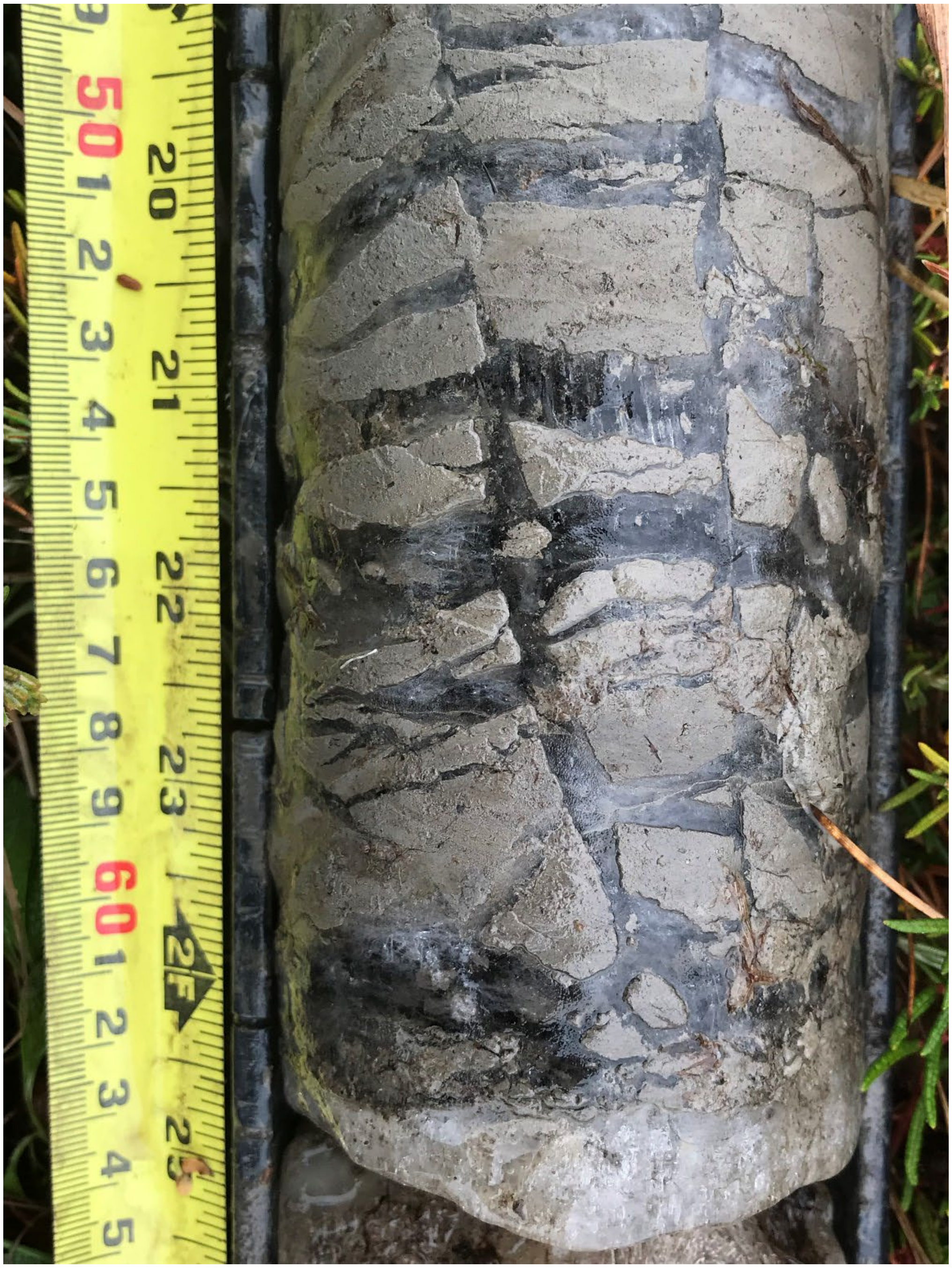

Figure S5. Ice-rich intermediate layer (53-61 cm) above wedge ice, silt, excess ice content 49.6% (54-60 cm); borehole AF-9, June 25, 2021; this borehole was drilled in the 60-cm-deep trough, water depth 8 cm. Occurrence of this layer indicates post-fire stabilization of partially degraded ice wedge.

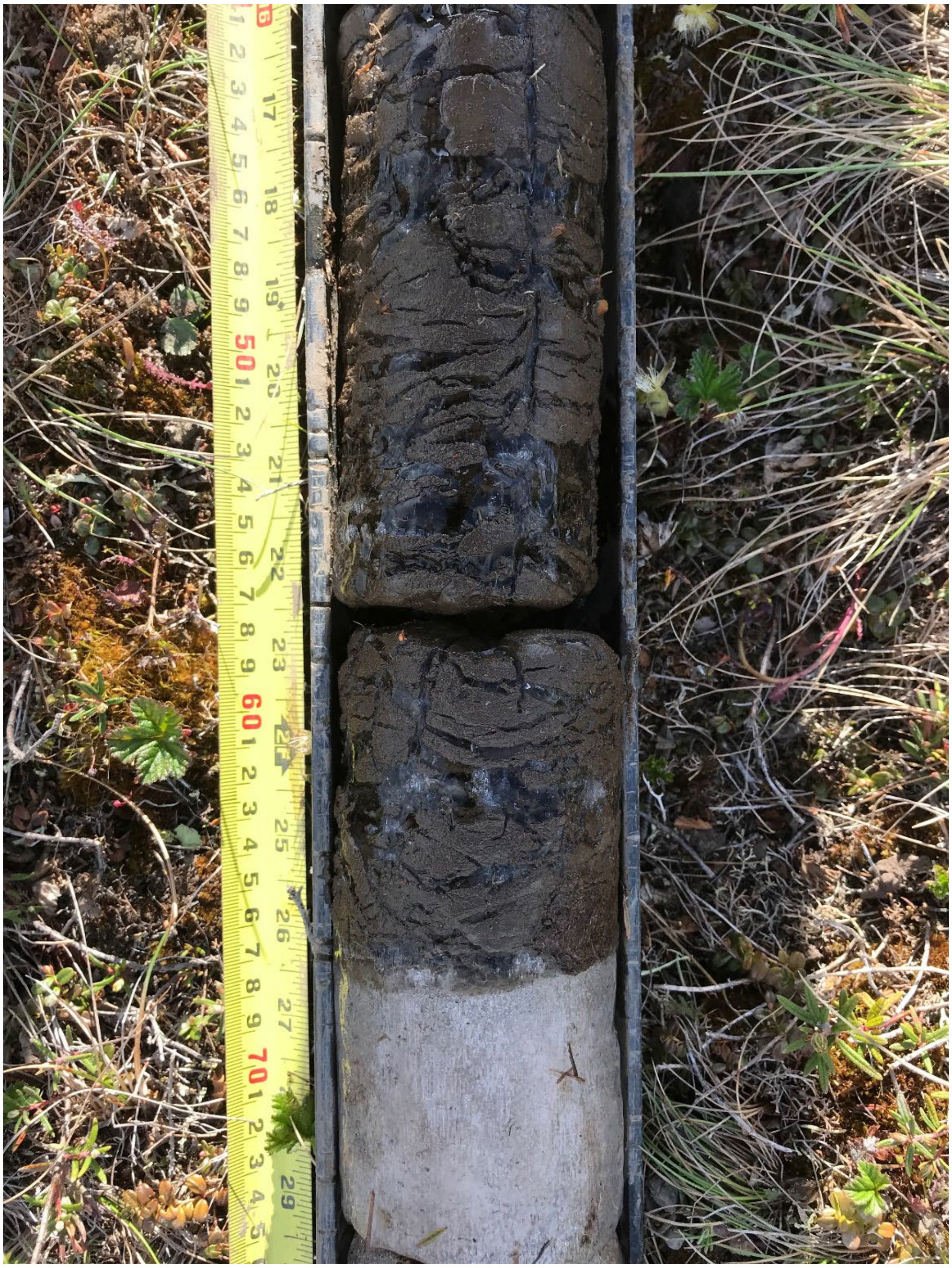

Figure S6. Ice-rich intermediate layer (40-66 cm) above wedge ice, silt, excess ice contents 46.2% (41-46 cm), 20.0% (53-62 cm); borehole AF-20, June 26, 2021; this borehole was drilled in the dry trough, approximately 5 cm above the water level. Occurrence of this layer indicates post-fire stabilization of partially degraded ice wedge.

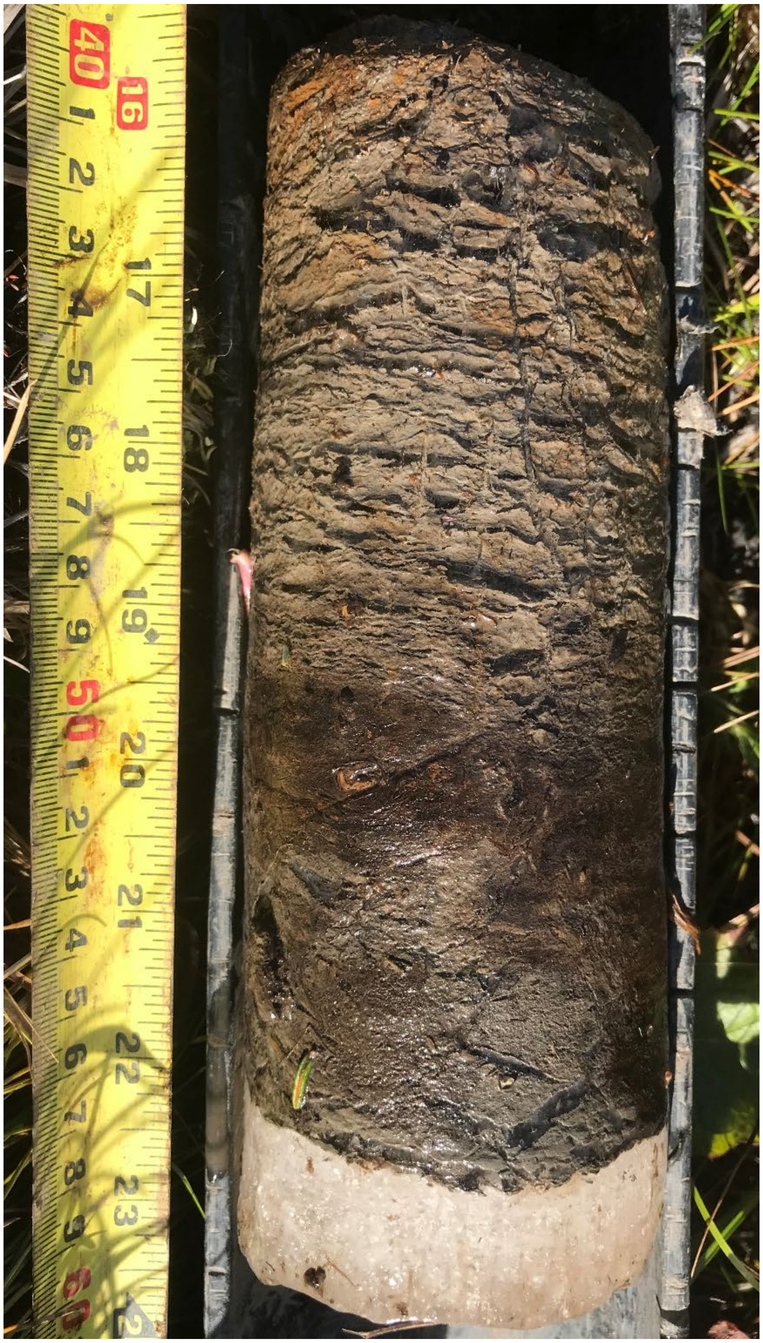

Figure S7. Ice-rich intermediate layer (41-56 cm) above wedge ice, silt with peat inclusions, with mainly reticulate cryostructure, excess-ice content 43.3% (41-49 cm); borehole AF-2/22, ice-wedge trough, yedoma surface, August 22, 2022. Occurrence of this layer indicates post-fire stabilization of partially degraded ice wedge.

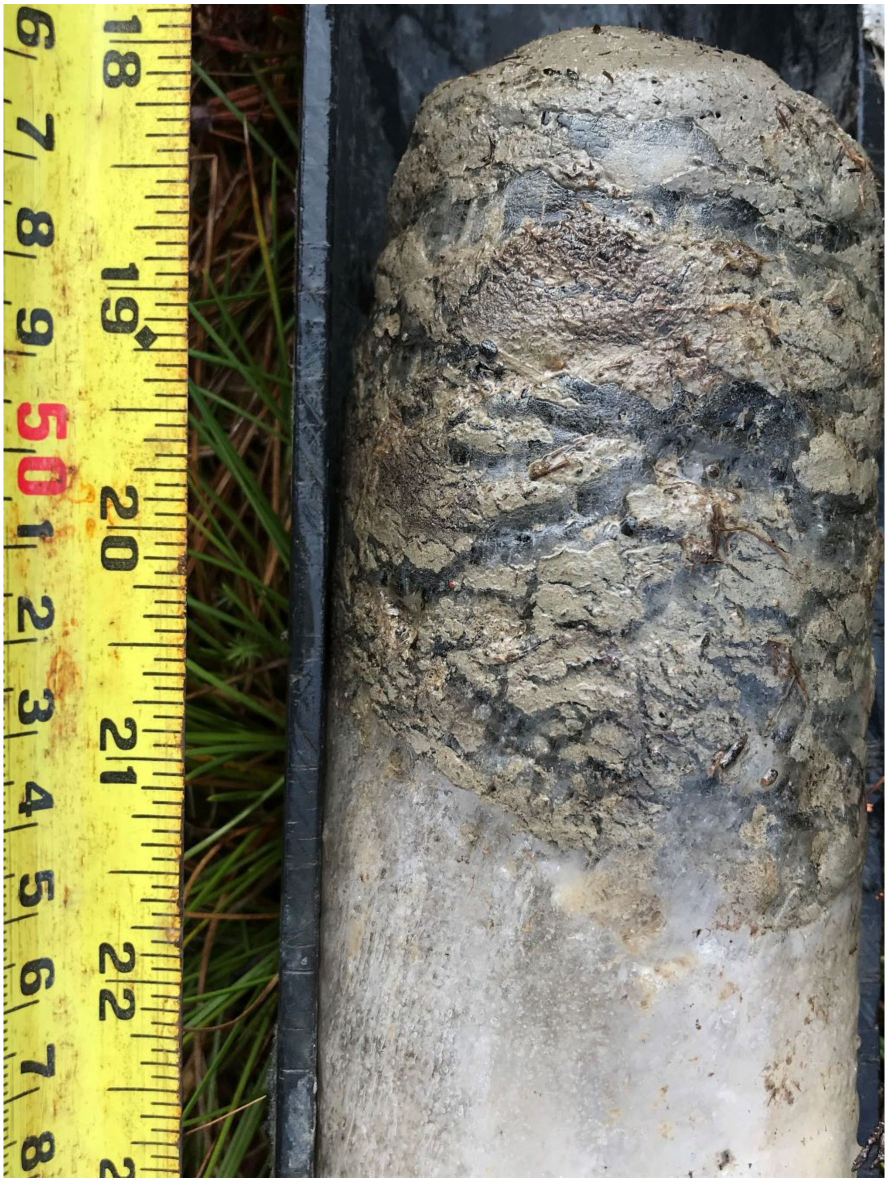

Figure S8. Ice-rich intermediate layer (44-55 cm) above wedge ice, silt with peat inclusions, ataxitic cryostructure, excess-ice content 26.1% (45-53 cm); borehole AF-5/22, water-filled ice-wedge trough with aquatic moss (water depth 30 cm), yedoma surface, August 22, 2022. Occurrence of this layer indicates post-fire stabilization of partially degraded ice wedge.

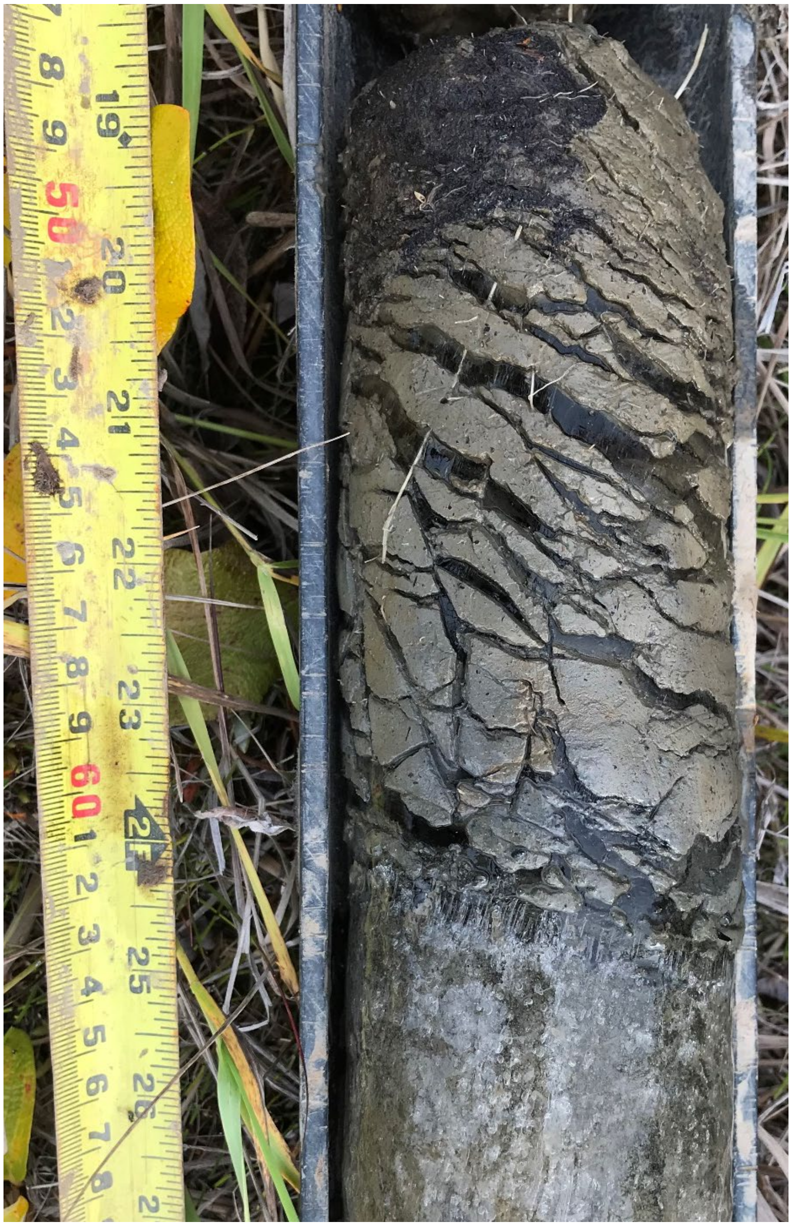

Figure S9. Ice-rich intermediate layer (51-61 cm) above wedge ice, silt with peat inclusions, mainly reticulate cryostructure, excess-ice content 38.8% (51-59 cm); borehole AF-23/22, dry ice-wedge trough, yedoma slope, August 24, 2022. Occurrence of this layer indicates post-fire stabilization of partially degraded ice wedge.

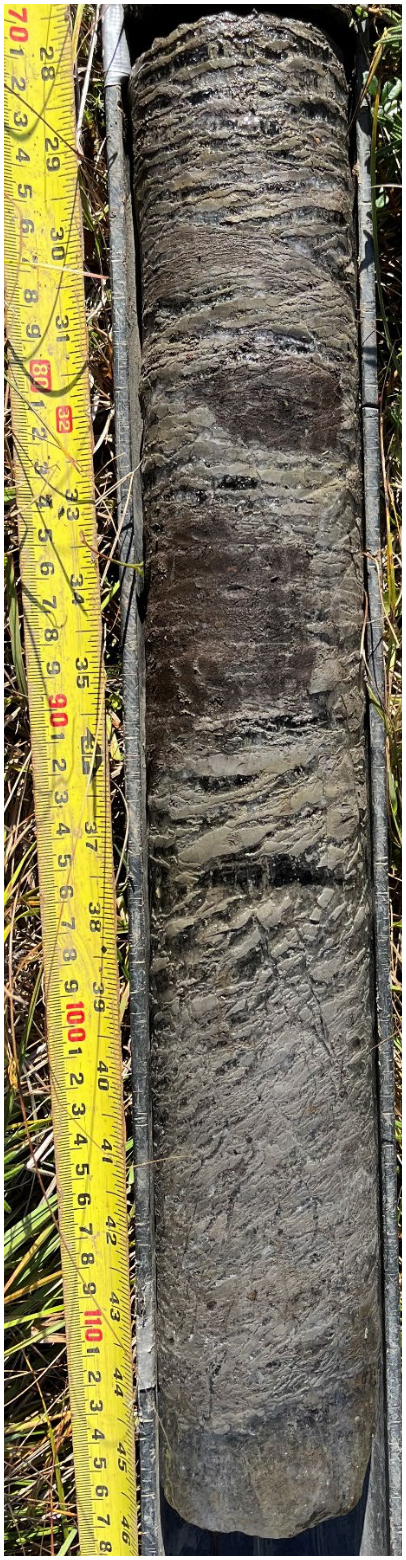

Figure S10. Ice-rich intermediate layer (65-111 cm) above the buried late Pleistocene ice wedge, silt with peat inclusions, excess-ice contents 45.1% (70-79 cm), 56.8% (100-110 cm); borehole ARF23-1, August 23, 2023, burned area, gentle yedoma slope, polygon center.

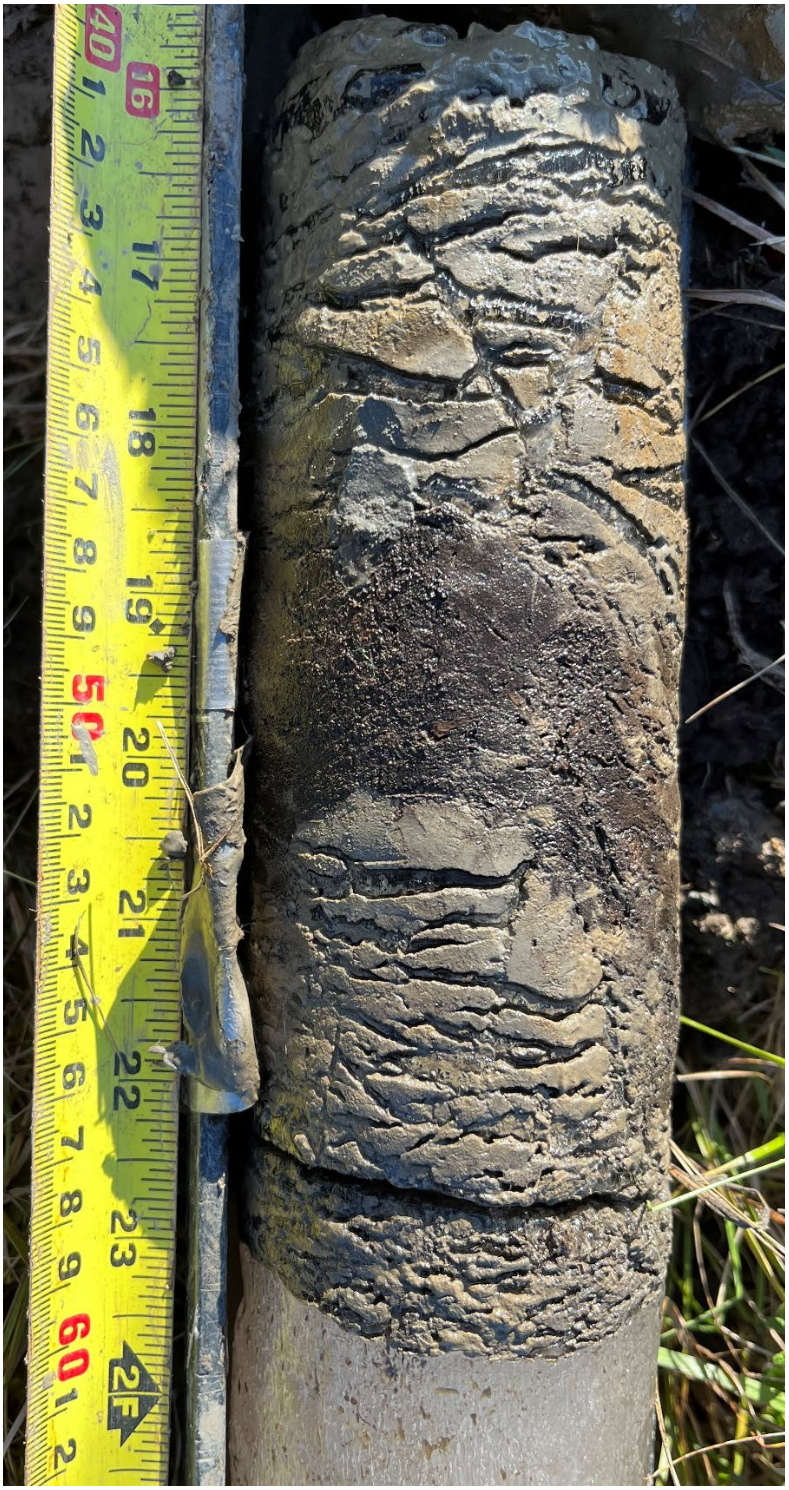

Figure S11. Recovered ice-rich intermediate layer (39-59 cm) above the recently degrading ice wedge, silt with peat inclusions, excess-ice content 28.0% (39-59 cm); borehole ARF23-2, August 23, 2023, burned area, gentle yedoma slope, center of the dry ice-wedge trough, ~250 cm wide, ~70 cm deep.

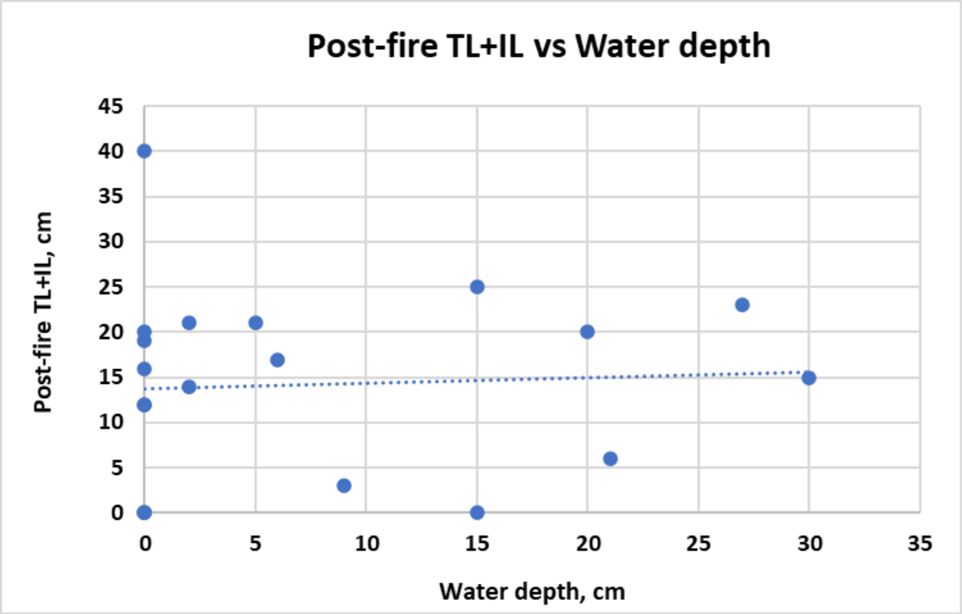

Figure S12. Thickness of the frozen protective layer (combined thickness of the post-fire transient and intermediate layers) with water depth, based in the drilling data in ice-wedge troughs in the Anaktuvuk River Fire study area, August 2022 and August 2023 (n=20).

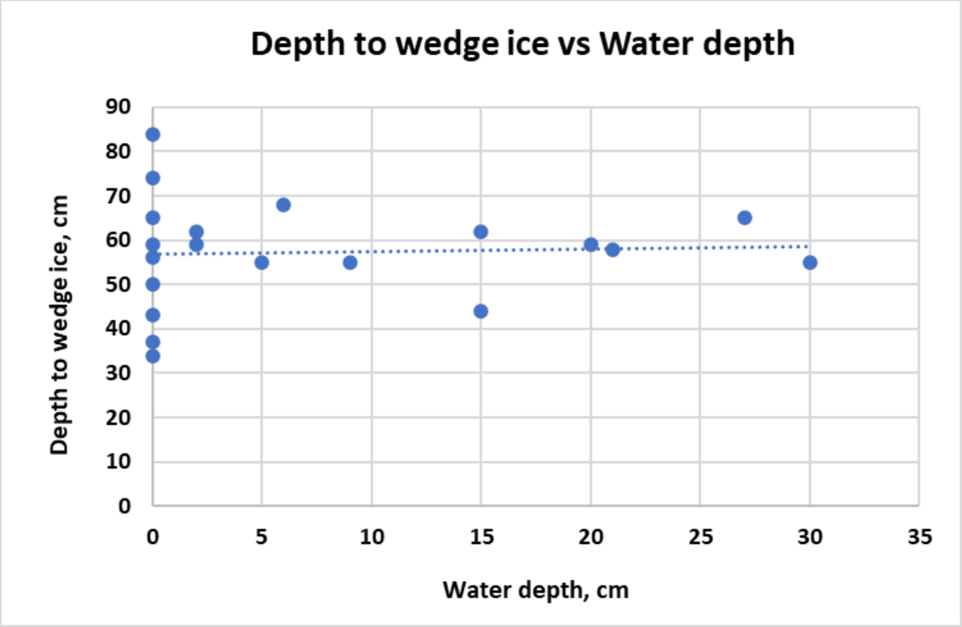

Figure S13. Depth to ice wedges with water depth, based in the drilling data in ice-wedge troughs in the Anaktuvuk River Fire study area, August 2022 and August 2023 (n=20).

Table S1. Cryostratigraphy and ground-ice content, Anaktuvuk River Fire study area, September 2010 (two areas: near AF-1 to AF-7 and near AF-15 to AF-20 2021 boreholes).

Borehole	Date	Coordinates	Depth, cm	Cryostratigraphic units	Sample depth, cm	GMC, %	VMC, %	EIC, %	Notes
**AL2-DOT**	9/1/10	69.25206 -<br>150.726791	0-57	ALU/moss, peat, silt					Burned area, yedoma<br>Main yedoma surface, Dry polygon center; polygons are hardly visible; thaw depths above adjacent degrading ice wedges 52-69 cm; troughs up to 250-300 cm wide, ~70 cm deep
			57-97	IL-WD/silt	57-75<br>83-97	222.59<br>123.82			
			97-600	IW					
**AL1-DOT**	9/1/10	69.26022<br>-150.70390	0-39	ALU/moss, peat, silt					Main yedoma surface, burned<br>~1.3 km from AL2-DOT;<br>Dry polygon center; burned tussocks
			39-73	IL-WD/silt	40-60	131.67			
			73-79	peat					
			79-145	IL-WD/silt	76-102<br>114-135	95.97<br>118.00			
			145-400	IW					
**AL2-1**	9/8/10	69.25185<br>-150.73398	0-12	ALU/burned peat					Main yedoma surface, burned<br>Dry polygon center; ~300 m from AL2-DOT<br>~1 m from AF-3
			12-42	ALU/silt					
			42-94	IL-WD/silt	43-52<br>75-84	104.18<br>86.95			
			94-150	IW					
**AL2-2**	9/8/10	69.25240<br>-150.73477	0-18	ALU/moss, peat					Main yedoma surface, burned<br>Dry polygon center; ~70 m from AL2-DOT
			18-43	ALU/peat					
			43-60	IL?/peat, silt					
			60-100	IL-WD/silt, peat	71-79<br>92-100	195.97<br>96.36			
			100-217	IL-WD/silt	122-131<br>157-166<br>192-200<br>209-217	79.58<br>124.97<br>87.15<br>155.68			
			217-250	IW Pleistocene?					
**AL2-3**	9/8/10	69.25125<br>-150.73357	0-15	ALU/moss, peat					Gentle yedoma slope, burned, closer to the lake<br>Polygon center; ~280 m from AL2-DOT;<br>Very wet surface
			15-49	ALU/silt					
			49-103	IL-WD/silt, peat	50-57<br>97-105	147.90<br>95.21			
			103-163	IL/silt	130-138<br>155-163	56.00<br>101.82			
			163-200	IW Pleistocene?					
**AL2-4**	9/9/10	69.25110<br>-150.73575	0-150	ALU/silt					Yedoma slope near the lake, burned area, top of the baydzherakh
			150-277	SP/silt (yedoma)	167-175<br>199-205<br>220-227<br>243-250<br>270-276	75.22<br>65.82<br>58.78<br>81.49<br>71.83			
**AL2-5**	9/9/10	69.25046<br>-150.72749	0-48	ALU/peat					Burned area, Thaw-lake basin,<br>Polygon center,<br>~7 m from AF-15
			48-137	SP/peat, silty peat	61-68<br>91-98<br>120-127	183.74<br>323.12<br>201.24			
			137-255	QSP-SP/silt, peat	148-157<br>185-193<br>211-219	168.66<br>144.65<br>83.64			
			255-289	PSP/silt	255-260<br>283-289	41.74<br>43.23			

			289-330	PSP/peat	325-330	233.83			
			330-389	PSP/silt	330-336<br>359-365<br>380-385	61.00<br>36.90<br>33.32			
**AL2-6**	9/9/10	69.25057<br>-150.72786	0-47	ALU/peat					Thaw-lake basin, burned area,<br>~4 m from AF-16<br>Trough ~150 cm wide, ~60 cm deep, ~10-15 cm above the water level
			47-110	IW					

Table S2. Cryostratigraphy and ground-ice content, Anaktuvuk River Fire study area, June 2021, **August 2022**, and August 2023.

**Borehole**	**Date**	**Coordinates**	**Depth, cm**	**Cryostratigraphic units**	**Sample depth, cm**	**GMC, %**	**VMC, %**	**EIC, %**	**Notes**
**ARFU**	6/23/21	69.228364,<br>-151.169080	0-11	ALU/moss, peat					Unburned area<br>Dry polygon center<br>IL>71 cm
			11-21	ALF/peat					
			21-25	ALF/silt					
			25-36	TL-IL-PD/silt, peat	25-33	147.80	76.66	4.28	
			36-107	IL-WD/silt, peat (50-70 – mostly peat)	40-50<br>65-72<br>79-87<br>98-107	252.50<br>196.39<br>127.78<br>227.67	84.87<br>81.36<br>76.56<br>85.33	48.49<br>1.11<br>30.58<br>60.22	
**ARFU-2**	6/23/21	69.228486,<br>-151.168997	0-12	ALU/moss, peat					Unburned area<br>11 m from ARFU<br>~4-m-wide trough, no water<br>IL>56 cm
			12-19	ALF/peat					
			19-29	ALF/peat, silt					
			29-40	ALF/silt	33-39	46.41	54.25	0.54	
			40-49	TL/silt	41-48	75.90	65.98	0.00	
			49-56	IL-PD/peat, silt					
			56-105	IL-WD/silt	58-65<br>75-84<br>97-105	132.99<br>358.11<br>165.88	77.27<br>90.15<br>82.45	50.87<br>78.45<br>63.54	
**AF-1**	6/24/21	69.25189217,<br>-150.733895	0-3	ALU/moss					Burned area<br>Near AL2-1<br>Dry polygon center<br>PFTD – 60 cm<br>TL = 8 cm<br>IL=0 cm<br>Post-fire permafrost aggradation = 8 cm
			3-11	ALU/silt					
			11-52	ALF/silt	35-40	22.51	38.93	0.00	
			52-60	TL/silt, peat					
			60-170	IL-WD/silt, peat incl	60-66<br>73-80<br>88-94<br>113-120<br>135-143<br>157-163	109.95<br>244.65<br>211.22<br>124.08<br>79.15<br>151.01	73.75<br>86.21<br>84.37<br>77.85<br>66.92<br>79.42	38.43<br>65.38<br>53.53<br>54.77<br>31.91<br>56.83	
			170-181	IW					
**AF-2**	6/24/21	69.25193431,<br>-150.7339468	0-5	ALU/moss, peat					Burned area<br>Near AL2-1<br>Wet trough, no water, ~70 cm deep<br>Belt at 30/32 cm<br>PFTD – 49 cm<br>TL = 0 cm<br>IL= 18 cm<br>Post-fire permafrost aggradation = 18 cm
			5-31	ALF/ silt, peat	17-24	51.24	56.70	0.00	
			31-49	TL-IL-PD/silt, peat	32-38<br>40-44	117.24<br>99.41	74.98<br>71.76	18.99<br>0.00	
			49-71	IW					
**AF-2/22**	8/22/22	69.25192632,<br>-150.7339741	0-30	ALU/peat					Near AF-2<br>Wet trough, water level<br>PFTD – 56 cm<br>TL = 4 cm
			30-37	ALU/ peat, silt					
			37-39	TL Destroyed core					
			39-41	TL silt					

			41-56	IL silt, peat	41-49	91.91	72.25	43.30	IL= 15 cm
			56-59	IW					Post-fire permafrost aggradation = 19 cm
**AF-3**	6/24/21	69.25184529	0-5	ALF/moss, peat					Burned area
		-150.7339998	5-11	ALF/peat, silt					Near AL2-1, 7 m from AF-1;
			11-15	ALF/silt					~1 m from AL2-1
			15-23	TL/silt	15-21	65.20	65.20	26.29	Water 13 cm, trough ~30-40 cm deep
			23-33	IL-PD/silt	24-30	89.96	89.96	26.16	Between the tussocks
			33-41	IL-WD/silt, peat incl	33-39	87.00	87.00	30.56	Belt at 33 cm
			41-52	IW					PFTD – 41 cm
									TL = 8 cm<br>IL= 18 cm<br>Post-fire permafrost aggradation = 26 cm
**AF-3/22**	8/22/22	69.25185492	0-11	ALU/peat					Near AF-3 (probably not very close)
		-150.7340818	11-34	ALU/ silt					Wet trough, no water
			34-74	TL-IL/silt, peat					51-54 cm – ataxitic, belt at 61 cm
			74-82	IW					PFTD – 74 cm
									TL = 17 cm<br>IL= 23 cm<br>Post-fire permafrost aggradation = 40 cm
**AF-4**	6/24/21	69.25185915	0-12	ALU/peat					Burned area
		-150.7341577	12-35	ALF/peat					Near AL2-1, same trench as AF-3
			35-47	TL silty peat					Wedge crossing
			47-55	IL-PD/org.silt	49-55	172.41	81.50	34.33	Water 12 cm, trough ~100 cm deep
			55-65	IL-PD/silt	59-65	89.88	69.67	31.91	Belt at 53/54 cm
			65-77	IW					PFTD – 65 cm
									TL = 12 cm<br>IL= 18 cm<br>Post-fire permafrost aggradation = 30 cm
**AF-4/22**	8/22/22	69.25186658	0-41	ALU/peat, silty peat					Near AF-4
		-150.7342313	41-62	TL-IL/peat, silt					Wedge crossing
			62-75	IW Pleistocene?					Water 2 cm
									PFTD – 62 cm<br>TL = 10 cm<br>IL= 11 cm<br>Post-fire permafrost aggradation = 21 cm
**AF-5**	6/24/21	69.25246956	0-18	ALU/mostly organic					Burned area
		-150.7348397	18-49	ALF/peat					Near AL2-2
			49-51	TL/silt					Water 24 cm, trough ~80-90 cm deep
			51-75	IW					No floating moss
									PFTD – 51 cm
									TL = 2 cm<br>IL= 0 cm<br>Post-fire permafrost aggradation = 2 cm
**AF-5/22**	8/22/22	69.25247418	0-40	ALU/mostly organic					Near AF-5
		-150.7347969	40-44	TL? Destroyed core					Water 30 cm, Aquatic moss
			44-55	IL/silt, peat	45-53	160.66	78.12	26.13	PFTD – 55 cm
			55-66	IW, Holocene?					TL = 4 cm
									IL= 11 cm<br>Post-fire permafrost aggradation = 15 cm
**AF-6**	6/24/21	69.25245153	0-16	ALU/peat					Burned area
		-150.7349316	16-34	ALF/peat, silty peat					Near AL2-2, the same trough as AF-5
			34-43	ALF/silt	34-40	46.16	54.12	0.00	Water 21 cm
			43-46	TL/silt					Aquatic moss
			46-56	IL-PD/silt	46-52	53.55	60.27	24.03	PFTD – 60 cm
			56-60	IL-WD/silt	56-60	105.66	74.96	50.73	TL = 3 cm
			60-79	IW Pleistocene?					IL= 14 cm
									Post-fire permafrost aggradation = 17 cm
**AF-6/22**	8/22/22	69.25244102	0-42	ALU/mostly peat					Near AF-6
		-150.734905	42-47	TL/peat, silt					Water 27 cm, Aquatic moss
			47-65	IL-PD/peat, silt					PFTD – 65 cm
			65-82	IW, Pleistocene?					TL = 5 cm

									IL= 18 cm<br>Post-fire permafrost aggradation = 23 cm
**AF-7**	6/24/21	69.25249792	0-11	ALU/mostly org					Burned area<br>Near AL2-2, the same trough as AF-5, AF-6<br>Dry trough ~40 cm deep<br>PFTD – 55 cm<br>TL = 0 cm<br>IL= 8 cm<br>Post-fire permafrost aggradation = 8 cm
		-150.7345229	11-36	ALF/peat					
			36-47	ALF-TL/silt, org	37-42	124.89	73.51	9.78	
			47-55	IL-PD/silt, org	47-53	172.26	79.29	24.23	
			55-86	IW					
**AF-7/22**	8/22/22	69.25250672	0-34	ALU/mostly org					Near AF-7<br>Water ~5 cm (average)<br>PFTD – 55 cm<br>TL = 10 cm<br>IL= 11 cm<br>Post-fire permafrost aggradation = 21 cm
		-150.7344918	34-55	TL-IL/peat, silt					
			55-66	IW, Holocene?					
**AF-8**	6/25/21	69.27067897	0-13	ALU/mainly organic					Burned area<br>Transect 37 m<br>Polygon center<br>Between the tussocks<br>PFTD – 62 cm<br>TL = 0 cm<br>IL= 17 cm<br>Post-fire permafrost aggradation = 17 cm
		-150.753491	13-45	ALF-TL/peat, silt	26-33	161.14	78.17	0.00	
			45-62	IL-PD/peat, silt	45-50	108.47	73.49	38.60	
			62-72	IL-WD/peat, silt	62-68	319.74	87.66	59.67	
			72-140	IL-WD/silt, peat	80-85<br>97-103<br>118-125<br>135-140	188.24<br>146.12<br>136.39<br>116.96	82.79<br>78.88<br>79.44<br>74.93	63.59<br>37.30<br>57.90<br>47.25	
			140-176	SP/silt	149-155<br>172-176	121.41<br>101.95	75.63<br>72.26	43.33<br>43.90	
			176-188	TCI+IW					
**AF-9**	6/25/21	69.27039127	0-14	ALU/peat, some silt					Burned area<br>Transect<br>Trough ~60 cm deep<br>Water 8 cm, Growing sedge<br>Belt at 53 cm<br>PFTD – 61 cm<br>TL = 10 cm<br>IL= 8 cm<br>Post-fire permafrost aggradation = 18 cm
		-150.7538038	14-17	ALF/peat					
			17-43	ALF/silt	34-39	28.00	44.24	0.66	
			43-53	TL-IL-PD/silt	44-50	50.12	58.68	13.74	
			53-61	IL-WD/silt	54-60	113.42	76.26	49.56	
			61-83	IW					
**AF-9/22**	8/23/22	69.27037387	0-52	ALU/peat, silt					Near AF-9<br>Water 9 cm, Growing sedge<br>PFTD – 55 cm<br>TL = 3 cm<br>IL= 0 cm<br>Post-fire permafrost aggradation = 3 cm
		-150.753796	52-55	TL/peat, silt					
			55-75	IW, Holocene?					
**AF-10**	6/25/21	69.27044369	0-24	ALU/mainly organic					Burned area<br>Transect<br>Trough ~70 cm deep<br>Wet, no water, growing sedge<br>PFTD – 82 cm<br>TL = 0 cm<br>IL= 31 cm<br>Post-fire permafrost aggradation = 31 cm
		-150.7537966	24-51	ALF/silt	40-45	24.42	40.89	0.00	
			51-64	TL-IL-PD/silt	55-61	56.52	61.55	24.29	
			64-82	IL-PD/silt	72-78	95.19	72.95	45.97	
			82-85	IW					
**AF-10/22**	8/23/22	69.27042586	0-14	ALU/peat					Near AF-10<br>Wet, no water; peat from 56/62 cm<br>PFTD – 65 cm<br>TL = 6 cm<br>IL= 6 cm<br>Post-fire permafrost aggradation = 12 cm
		-150.7537983	24-53	ALU/silt w/clay					
			53-65	TL-IL/silt, peat					
			65-73	IW, Holocene					
**AF-11**	6/25/21	69.27054281	0-27	ALU/silt, org on top					Burned area<br>Transect<br>Trough ~60 cm deep<br>Water 14 cm, near the edge of the pond<br>PFTD – 63 cm<br>TL = 3 cm<br>IL= 10 cm
		-150.7533743	27-50	ALF/silt, peat					
			50-53	TL/silt, org	48-53	58.20	59.80	0.00	
			53-63	TL-IL-PD/silt, org	55-60	114.41	71.77	6.91	
			63-69	IW					

									Post-fire permafrost aggradation = 13 cm
**AF-11/22**	8/23/22	69.27054517<br>-150.7533827	0-52	ALU/org, silt w/clay					Near AF-11<br>Water 21 cm, near the edge of the pond<br>PFTD – 58 cm<br>TL = 6 cm<br>IL= 0 cm<br>Post-fire permafrost aggradation = 6 cm
			52-58	TL/peat, silt					
			58-80	IW, Holocene					
**AF-12**	6/25/21	69.27023527<br>-150.7516327	0-12	ALU/organic					Burned area<br>Transect 14 m, upper slope<br>Wet trough, ~80 cm deep, no water<br>PFTD – 54 cm<br>TL = 0 cm<br>IL= 16 cm<br>Post-fire permafrost aggradation = 16 cm
			12-38	ALF/peat, silt					
			38-54	TL-IL-PD/silt, peat	44-49	132.81	77.24	34.06	
			54-78	IW					
**AF-12/22**	8/23/22	69.2702366<br>-150.7516891	0-68	ALU					Near AF-12<br>no water<br>PFTD – 84 cm<br>TL = 8 cm<br>IL= 8 cm<br>Post-fire permafrost aggradation = 16 cm
			68-84	TL-IL-PD/peat, silt					
			84-95	IW, Pleistocene					
**AF-13**	6/25/21	69.270528<br>-150.7523685	0-12	ALU/mainly mineral					Burned area<br>Transect 25 m, upper slope<br>Dry trough<br>PFTD – 79 cm<br>TL = 0 cm<br>IL= 16 cm<br>Post-fire permafrost aggradation = 16 cm
			12-38	ALF/silty clay					
			38-63	ALF/silt					
			63-79	IL-PD/silt					
			79-103	IW Pleist-Holocene?					
**AF-13/22**	8/23/22	69.27051264<br>-150.7523437	0-23	ALU/peat					Near AF-13<br>Wet trough<br>PFTD – 50 cm<br>TL = 12 cm<br>IL= 0 cm<br>Post-fire permafrost aggradation = 12 cm
			23-38	ALU/org-min					
			38-46	TL/peat					
			46-50	TL/silt					
			50-58	IW Pleist-Holocene?					
**AF-14**	6/25/21	69.27051126<br>-150.7530228	0-10	ALU/silty clay, org					Burned area<br>Transect 2.5 m, near the slope<br>Polygon center<br>PFTD – 59 cm<br>TL = 4 cm<br>IL= 17 cm<br>Post-fire permafrost aggradation = 21 cm
			10-31	ALF-TL/peat					
			31-38	ALF/silt, peat					
			38-42	TL/silt					
			42-59	IL-PD/silt	47-53	80.45	69.50	43.93	
			59-130	IL-WD/silt	61-66<br>76-84<br>109-115	140.12<br>130.12<br>155.07	79.88<br>78.66<br>81.46	61.93<br>54.62<br>62.16	
**AF-14/22**	8/23/22	69.27050917<br>-150.753051	0-23	ALU/peat					Near AF-14<br>Polygon center<br>Belt at 54 cm<br>PFTD – 66 cm<br>TL = 12 cm<br>IL= 12 cm<br>Post-fire permafrost aggradation = 24 cm
			23-42	ALU/org.silt					
			42-54	TL/peat, silt					
			54-66	IL-PD/silt					
			66-112	IL-WD/silt					
**AF-15**	6/26/21	69.25045969<br>-150.7277087	0-15	ALU/moss, peat					Burned area<br>DTLB, near AL2-5,6 ~7 m from AL2-5<br>Polygon center, LCP<br>Water 5 cm<br>PFTD – 52 cm<br>TL = 16 cm<br>IL= 0 cm<br>Post-fire permafrost aggradation = 16 cm
			15-36	ALF/peat, org silt	25-30	100.00	71.88	0.00	
			36-52	TL/org. silt, peat	36-40<br>47-52	101.35<br>195.74	72.15<br>81.31	1.92<br>0.00	
			52-157	SP/peat, org. silt	58-64<br>125-131	576.88<br>442.68	90.58<br>88.07	43.38<br>23.96	
			157-180	QSP-SP/silt, peat	171-177	105.70	72.98	36.47	
			180-202	PSP/silt, peat	190-195	71.64	64.67	26.18	
**AF-16**	6/26/21	69.25056217<br>-150.7277645	0-4	ALU/org					Burned area, ~12m N of AF-15,~4m from AL2-6;<br>Trough ~240 cm wide, pond ~140 cm wide, rims ~60 cm above the water<br>Water 44 cm
			4-32	ALF/peat					
			32-56	IL-WD/peat					
			56-62	IL-WD/silt, org-rich	56-62	574.43	92.73	65.05	

			62-96	IW very clean					Belts 32-38 cm combined<br>PFTD – 62 cm<br>TL = 0 cm<br>IL= 30 cm<br>Post-fire permafrost aggradation = 30 cm
**AF-16/22**	8/22/22	69.25056799<br>-150.7278092	0-17	ALU/org					Near AF-16<br>Water 40 cm, Aquatic moss<br>Didn't drill (PL2 is probably ~45 cm)
**AF-17**	6/26/21	69.25056933<br>-150.7276819	0-31	ALU/moss, peat					Burned area, same trough as AF-16<br>Trough, ~200 cm wide, rims ~40 cm<br>Wet, water <10 cm (at places), thick moss<br>Several thin belts at 50-56 cm<br>No samples<br>PFTD – 66 cm<br>TL = 0 cm<br>IL= 16 cm<br>Post-fire permafrost aggradation = 16 cm
			31-50	ALF/peat					
			50-66	IL-PD/peat					
			66-73	IW					
**AF-17/22**	8/22/22	69.25056039<br>-150.7277113	0-51	ALU/moss, peat					Near AF-17<br>Water 6 cm<br>Belt at 55 cm<br>PFTD – 68 cm<br>TL = 4 cm<br>IL= 13 cm<br>Post-fire permafrost aggradation = 17 cm
			51-55	TL/peat					
			55-68	IL/mostly peat					
			68-72	IW					
**AF-18**	6/26/21	69.250604<br>-150.727503	0-13	ALU/moss, sedge					Burned area, near AF-17 - adjacent trough<br>Trough ~220 cm, pond ~170 cm, rims ~40 cm (above the water level)<br>Water 18 cm<br>PFTD – 64 cm<br>TL = 0 cm<br>IL= 19 cm<br>Post-fire permafrost aggradation = 19 cm
			13-38	ALF-TL/peat					
			38-45	ALF-TL/org silt					
			45-64	IL-PD/org silt	44-52	215.05	82.69	24.87	
			64-85	IW					
**AF-18/22**	8/22/22	69.250594<br>-150.727504	0-39	ALU/moss, peat					Near AF-18<br>Water 20 cm<br>PFTD – 59 cm<br>TL = 0 cm<br>IL= 20 cm<br>Post-fire permafrost aggradation = 20 cm
			39-46	IL-WD/silt, org					
			46-59	IL-PD/peat					
			59-71	IW					
**AF-19**	6/26/21	69.25063699<br>-150.7274532	0-16	ALU/moss, peat					Burned area, same trough as AF-18<br>Dry trough, ~160 cm wide, rims ~15-20 cm,<br>Elevated part of the trough, ~10-15 cm above the water level<br>No samples<br>PFTD – 42 cm<br>TL = 6 cm<br>IL= 0 cm<br>Post-fire permafrost aggradation = 6 cm
			16-36	ALF/org silt					
			36-42	TL/silt					
			42-59	IW					
**AF-19/22**	8/22/22	69.25064848<br>-150.7274231	0-43	ALU/peat, silt					0.5 m from AF-19, elevated part of the trough<br>Degrading ice wedge<br>PFTD – 43 cm<br>TL = 0 cm<br>IL= 0 cm<br>Post-fire permafrost aggradation = 0 cm
			43-69	IW, Holocene					
**AF-20**	6/26/21	69.25072773<br>-150.7271863	0-23	ALU/moss, peat					Burned area, same trough as AF-19<br>Dry trough, ~ 5 cm above the water level, ~130 cm wide; rims ~20 cm high<br>Belts from 46 cm<br>PFTD – 66 cm<br>TL = 0 cm<br>IL= 26 cm<br>Post-fire permafrost aggradation = 26 cm
			23-30	ALF/peat					
			30-40	ALF/silt w/clay	33-38	49.76	55.98	0.00	
			40-46	IL-PD/silt	41-46	159.54	80.30	46.16	
			46-66	IL-WD/silt	53-62	95.64	70.97	20.02	
			66-88	IW					
**AF-20/22**	8/22/22	69.25070831	0-25	ALU/moss, peat					0.5 m from AF-19

		-150.7272333	25-45	ALU/silty peat					Water 2 cm<br>Belts from 52 cm<br>PFTD – 59 cm<br>TL = 7 cm<br>IL= 7 cm<br>Post-fire permafrost aggradation = 14 cm
			45-59	IL/silt, peat					
			59-62	IW, Holocene					
**AF-21**	6/27/21	69.24693103<br>-150.718951	0-13	ALU/moss					Unburned area, yedoma slope, ~ 2 m lower than the main surface, top of the poorly developed baydzherakh<br>IL=0 cm<br>No samples
			13-22	ALF/peat					
			22-38	ALF/silt, peat					
			38-47	TL/silt, peat					
			47-75	IW Pleist-Holocene?					
**AF-22**	6/27/21	69.246882<br>-150.718711	0-4	ALU/moss					Unburned area, yedoma slope, ~ 0.8-1 m lower than AF-21, top of the poorly developed baydzherakh<br>IL=17 cm
			4-15	ALU/silt					
			15-36	ALF/silt, peat					
			36-41	TL silt	36-41	59.21	60.21	11.25	
			41-58	IL-WD/silt	41-53	97.62	73.44	47.43	
			58-99	SP/silt (yedoma)	73-79<br>94-99	77.19<br>95.81	66.36<br>71.00	28.40<br>40.35	
**AF-23**	6/27/21	69.24684796<br>-150.718925	0-18	ALU/moss					Unburned area, yedoma slope, ~ 2 m lower than AF-22, depression between baydzherakhs<br>Water 7 cm<br>IL=12 cm
			18-32	ALF/silt, peat					
			32-38	ALF/peat					
			38-44	TL-IL-PD/silt, peat					
			44-50	IL-WD/silt	44-50	190.24	82.94	56.77	
			50-71	IW Pleist-Holocene?					
**AF-23/22**	8/24/22	69.24686484<br>-150.719023	0-20	ALU/moss, peat					Unburned area, yedoma slope,<br>Near AF-23<br>No water (relatively dry depression)<br>IL=10 cm<br>PL2=16 cm
			20-45	ALU/silt					
			45-51	TL/silt, peat					
			51-61	IL-WD/silt	51-59	69.61	66.35	38.76	
			61-71	IW Pleistocene?					
**AF-24**	6/27/21	69.24695476<br>-150.7194301	0-8	ALU/moss, peat					Burned area, yedoma slope, top of the baydzherakh<br>PFTD – 52 cm<br>TL = 8 cm<br>IL= 0 cm<br>Post-fire permafrost aggradation = 8 cm
			8-14	ALF/peat					
			14-44	ALF/silt	40-44	30.33	46.22	0.00	
			44-52	TL/silt					
			52-94	SP/silt (yedoma)	63-67<br>90-94	76.71<br>69.36	68.49<br>63.93	36.71<br>26.86	
**AF-25**	6/27/21	69.246981<br>-150.7193859	0-4	ALU/moss, peat					Burned area, yedoma slope,<br>Trough near AF-24, ~1 m lower<br>Water 10 cm<br>No samples<br>PFTD – 31 cm<br>TL = 7 cm<br>IL= 0 cm<br>Post-fire permafrost aggradation = 7 cm
			4-9	ALF/peat					
			9-24	ALF/silt, peat					
			24-31	TL/silt					
			31-70	IW Pleistocene?					
**AF-25/22**	8/24/22	69.24698073<br>-150.719462	0-22	ALU/moss, peat					Near AF-25, degrading ice wedge<br>Wet, no water<br>PFTD – 34 cm<br>TL = 0 cm<br>IL= 0 cm<br>Post-fire permafrost aggradation = 0 cm
			22-34	ALU/silt, peat					
			34-67	IW Pleistocene					
**AF-25A/22**	8/24/22	69.24698073<br>-150.7194787	0-3	ALU peat					~0.6 m from AF-25/22, degrading ice wedge<br>Dry center of the frost boil<br>PFTD – 59 cm<br>TL = 0 cm<br>IL= 0 cm<br>Post-fire permafrost aggradation = 0 cm
			3-50	ALU/silt, org					
			50-59	ALU/peat					
			59-88	IW Pleistocene					
**AF-26**	6/27/21	69.24690877<br>-150.719502	0-14	ALU/moss					Burned area, yedoma slope<br>Trough ~470 cm wide, near AF-25, ~1.5-2 m lower<br>Wet, no water
			14-26	ALF/silt, org					
			26-29	TL/silt					
			29-34	IL-PD/silt	29-34	89.65	69.62	26.39	

			34-60	IW Pleistocene?					Belt at 29 cm<br>PFTD – 34 cm<br>TL = 0 cm<br>IL= 0 cm<br>Post-fire permafrost aggradation = 0 cm
**AF-26/22**	8/24/22	69.2469102<br>-150.7195919	0-10	ALU/moss, peat					Near AF-26, degrading ice wedge<br>Wet, no water<br>PFTD – 37 cm<br>TL = 0 cm<br>IL= 0 cm<br>Post-fire permafrost aggradation = 0 cm
			10-24	ALU/silty peat					
			24-37	ALU/silt, peat					
			37-60	IW Pleistocene					
**AF-27**	6/27/21	69.24688929<br>-150.7196143	0-7	ALU/moss, peat					Burned area, yedoma slope<br>Trough, ice-wedge crossing, near AF-26, ~1.3 m lower<br>Water 12 cm<br>No samples<br>PFTD – 42 cm<br>TL = 4 cm<br>IL= 0 cm<br>Post-fire permafrost aggradation = 4 cm
			7-33	ALF/peat					
			33-38	ALF/silt					
			38-42	TL/silt					
			42-68	IW Pleistocene?					
**AF-27/22**	8/24/22	69.24686986<br>-150.7196574	0-44	ALU/mostly org.					Near AF-27, degrading ice wedge<br>Water 15 cm<br>PFTD – 44 cm<br>TL = 0 cm<br>IL= 0 cm<br>Post-fire permafrost aggradation = 0 cm
			44-68	IW Pleistocene					
**AF-28**	6/27/21	69.24728289<br>-150.7191233	0-3	ALU/moss					Burned area, main yedoma surface, polygon center, not far from AF-27<br>PFTD – 87 cm<br>TL = 12 cm<br>IL= 27 cm<br>Post-fire permafrost aggradation = 39 cm
			3-20	ALU/silt					
			20-28	ALF/silt					
			28-38	ALF/peat					
			38-48	ALF/silt, peat					
			48-60	TL/silt, peat					
			60-87	IL-PD/silt, peat	68-73	119.65	75.36	48.96	
			87-106	IL-PD/silt, peat	94-100	164.74	80.81	60.97	
			106-124	SP/silt (yedoma)	119-124	148.01	79.09	47.63	
**AF-29**	6/27/21	69.247317<br>-150.71945	0-6	ALU/moss, peat					Burned area, main yedoma surface<br>Trough ~180 wide, ~150 deep, adjacent to AF-28,<br>Water 21 cm<br>No samples<br>PFTD – 44 cm<br>TL = 0 cm<br>IL= 0 cm<br>Post-fire permafrost aggradation = 0 cm
			6-27	ALF/silt					
			27-44	ALF-TL/peat					
			44-84	IW Pleist-Holocene?					
**AF-29/22**	8/24/22	69.24734121<br>-150.7194088	0-25	ALU/peat					Near AF-29<br>Water 15 cm<br>PFTD – 62 cm<br>TL = 9 cm<br>IL= 16 cm<br>Post-fire permafrost aggradation = 25 cm
			25-37	ALU/silt					
			37-46	TL/silt, peat					
			46-62	IL-PD/silt, peat	46-55	106.75	73.18	9.32	
			62-67	IW Holocene?					
**ARF23-1**	8/23/23	69.27697164<br>-150.7175424	0-8	ALU/peat					Burned area, gentle upper yedoma slope, polygon center<br>~0.5 m from the datalogger<br>PFTD – 65 cm<br>TL = 6 cm<br>IL= 7 cm<br>Post-fire permafrost aggradation = 13 cm
			8-52	ALU/peat					
			52-65	TL-IL/silt	55-65	142.14	78.41	38.28	
			65-111	IL-WD/silt	70-79<br>100-110	88.86<br>135.65	71.57<br>79.35	45.12<br>56.75	
			111-138	IW					
			138-146	QSP/silt	136-146	141.18	78.30	51.75	
**ARF23-2**	8/23/23	69.27705691<br>-150.7176722	0-16	ALU/moss, peat					Burned area, Dry trough, ~250 cm wide, ~70 cm deep<br>~8 m from ARF23-1<br>PFTD – 59 cm
			16-39	ALU/silt					
			39-59	IL/silt, peat	39-49	115.40	74.68	28.03	
			59-102	IW, Holocene					

									TL = 0 cm<br>IL= 20 cm<br>Post-fire permafrost aggradation = 20 cm

ABBREVIATIONS (Tables S1 and S2):

Cryostratigraphic units
ALU – unfrozen active layer
ALF – frozen AL (ice-poor; often with dry friable soil horizons closer to the base of the AL)
TL – transient layer (relatively ice-poor, mainly with reticulate and/or braided cryostructures)
ALF-TL – undifferentiated AL/TL (no distinctive boundary between AL and TL)
IL-WD – intermediate layer, well developed (thick belts, mainly ataxitic cryostructure, EIC >30-40%)
IL-PD – intermediate layer, poorly developed (relatively ice-poor, no well-developed belts)
TL-IL – undifferentiated TL/IL (no distinctive boundary between TL and IL)
SP – syngenetic permafrost (thin belts, micro-cryostructures)
QSP – quasi-syngenetic permafrost (buried intermediate layer)
PSP – para-syngenetic permafrost (refrozen talik)

Ground-ice contents
GMC – gravimetric moisture content, % wt
VMC – volumetric moisture content, % vol
EIC – excess-ice content, % vol

Massive ice
IW – ice wedge
TCI – thermokarst-cave ice

Notes Abbreviations:

PFTD – post-fire thaw depth, cm (max)
TL – transient layer, cm
IL – Intermediate layer, cm
Post-fire permafrost aggradation – TL+IL, cm
